# Suppression of Ciliogenesis Alleviates Cellular Senescence via AKT Signaling in Gingival Aging

**DOI:** 10.1111/acel.70627

**Published:** 2026-07-10

**Authors:** Wenjun Shao, Huihui Yang, Chenghu Yin, Yunjie Zhang, Yixing Xu, Wakam Chang, Haibin Xia, Min Wang, Liangliang Fu, Kaiyao Huang

**Affiliations:** ^1^ State Key Laboratory of Oral & Maxillofacial Reconstruction and Regeneration, Key Laboratory of Oral Biomedicine Ministry of Education, Hubei Key Laboratory of Stomatology, School & Hospital of Stomatology Wuhan University Wuhan China; ^2^ Department of Nephrology, Wuhan Children's Hospital (Wuhan Maternal and Child Healthcare Center), Tongji Medical College Huazhong University of Science & Technology Wuhan China; ^3^ Key Laboratory of Algal Biology, Institute of Hydrobiology Chinese Academy of Sciences Wuhan China; ^4^ Department of Biomedical Sciences, Faculty of Health Sciences University of Macau Macau China; ^5^ Department of Oral Implantology, School and Hospital of Stomatology Wuhan University Wuhan China

**Keywords:** aging, cellular senescence, fibroblasts, gingiva, periodontal diseases, primary cilia

## Abstract

Aging, as an intrinsic risk factor, accelerates gingival inflammation and periodontal diseases. However, the cellular and molecular mechanisms underlying gingival aging remain unclear, hindering the development of targeted therapies. In this study, we performed the first single‐cell transcriptomic analysis of aging human gingiva, identifying primary cilia as potential regulators of gingival fibroblast senescence. We demonstrated that aged gingival tissues exhibit increased fibroblast senescence and enhanced ciliogenesis compared to young tissues. Suppression of ciliogenesis significantly reduced senescence markers and alleviated DNA damage in aged fibroblasts accompanied by increased AKT activation. In addition, FOXO1 inactivation and enhanced expression of DNA repair‐related genes were found after suppression of ciliogenesis in aged gingival fibroblasts. Importantly, inhibition of AKT partially reversed the anti‐senescent phenotypes induced by ciliogenesis suppression. Functionally, adeno‐associated virus‐mediated suppression of ciliogenesis in aged mice mitigated gingival fibroblast senescence, reduced inflammation, and diminished tissue fibrosis. These findings highlight that primary cilia may contribute to the regulation of gingival fibroblast senescence and identify ciliary dynamics and AKT signaling downstream of cilia as potential therapeutic targets for managing aging‐related gingival diseases, providing a potential strategy to improve periodontal health in the elderly.

## Graphical abstract

In human gingival aging, aged gingival fibroblasts show increased senescence with enhanced primary cilia formation. Targeting cilia formation by knocking down IFT88 could reverse fibroblast senescence via the AKT signaling and alleviate periodontal inflammation.

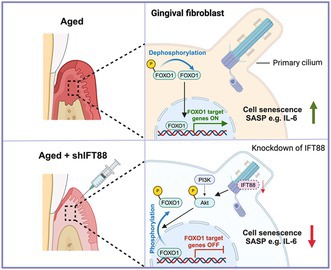

## 1 Introduction

Aging is a universal and multifaceted process that significantly affects human health, contributing to the onset and progression of various chronic diseases. Among these, periodontal diseases are notably prevalent among older adults, with more than 60% of individuals over 65 years suffering from varying degrees of gingival inflammation and tissue degradation (Eke et al. 2020). Periodontal diseases also elevate the risk of systemic conditions, such as cardiometabolic, cognitive neurodegenerative, and autoimmune diseases (Hodjat et al. 2020; Williams et al. 2021), highlighting the critical role of the gingiva in linking periodontal and whole‐body health. The gingiva serves as the first line of defense in periodontal tissues against microbial invasion and physicochemical stimuli, and disruption of its immune microenvironment significantly increases susceptibility to periodontitis (Aquino‐Martinez 2023; Hu et al. 2024). Therefore, gingiva aging contributes significantly to the occurrence of periodontal diseases in the elderly; however, the molecular mechanisms underpinning gingival aging remain poorly understood, posing a major challenge for developing targeted therapies.

The gingiva, part of the oral mucosa surrounding the teeth and covering the alveolar bone, consists of two main layers: the surface squamous epithelium and the deeper lamina propria. The lamina propria, a connective tissue, is dominated by gingival fibroblasts (GFs). Clinical comparisons of gingival tissues from healthy individuals and patients with periodontitis showed that macrophages and fibroblasts were profoundly affected by cellular senescence (Rattanaprukskul et al. 2024). An snRNA‐seq study in aging cynomolgus monkeys also identified senescence characteristics in GFs (Hu et al. 2024). Disordered responses of GFs to extracellular signals have also been shown to aggravate chronic inflammation by producing cytokines or inflammatory mediators (Wielento et al. 2023). It is reported that fibroblasts are key structural cells that regulate gingival tissue homeostasis, and they drive the progression of periodontitis by promoting neutrophil and leukocyte recruitment via intercellular signaling (Williams et al. 2021). These findings suggest that fibroblasts are a central component of gingival structural immunity, and are essential for maintaining periodontal homeostasis.

With aging, the accumulation of senescent cells becomes a key factor driving the disruption of gingival immune homeostasis (Di Micco et al. 2021; Hu et al. 2024). Our previous work has demonstrated that senescent gingival fibroblasts activate neutrophil extracellular trap (NET) formation and macrophage M1 polarization via SASP factors, resulting in structural and immune homeostasis disruption, thereby exacerbating periodontal inflammation (Guo et al. 2024). Moreover, our further experiments demonstrated that pharmacologically targeting senescent fibroblasts can alleviate gingival inflammation and reduce destruction of both soft and hard periodontal tissues (Fu et al. 2025; Yin et al. 2025). This suggests that fibroblast senescence may be a key factor driving structural immune imbalance in the gingiva. Therefore, thorough exploration of the mechanisms driving fibroblast aging will help identify specific targets in senescent fibroblasts, providing novel therapeutic strategies to restore control over periodontitis progression.

Primary cilia, acting as the antenna of cells including GFs, sense and transmit extracellular signals to modulate cell fate (Wang et al. 2021). Genetic screens in both mouse stem cells and *Caenorhabditis elegans* showed strong correlation between cilia and aging (Apfeld and Kenyon 1999; Ruetz et al. 2024). Furthermore, cilia‐mediated signaling pathways including Sonic Hedgehog (Shh), Wnt, and Notch signaling are all disturbed during brain aging (Inestrosa et al. 2020; Ma et al. 2022; Sun et al. 2013). Therefore, cilia or cilia‐mediated signaling pathways provide a potential target for anti‐aging therapy. In addition, ciliopathies caused by ciliary defects or dysfunction (such as Ellis‐van Creveld syndrome and oral‐facial‐digital syndrome) are sometimes accompanied by disrupted odontogenesis or oral functional abnormalities (Hampl et al. 2017). Primary cilia are reported to be promising regulators in some oral and maxillofacial diseases, such as tumors and malocclusion (Liu et al. 2024). Thus, drug therapies targeting primary cilia or their signaling pathways could provide novel perspectives for managing oral diseases.

In this study, we address the critical knowledge gap by employing single‐cell RNA sequencing (scRNA‐seq) to chart the cellular landscape of aged human gingiva, with a focus on primary cilia of GFs. We also investigate the functional consequences of ciliary inhibition on GF senescence through in vitro and in vivo models and identify the AKT signaling pathway as a key mechanistic link. Our findings uncover primary cilia as promising therapeutic targets for attenuating gingival fibroblast senescence and mitigating gingival aging, presenting new strategies for managing periodontitis in the elderly population.

## 2 Results

### 2.1 Aged Human Gingival Tissues Display Features of Cellular Senescence and Chronic Inflammation

**FIGURE 1:**
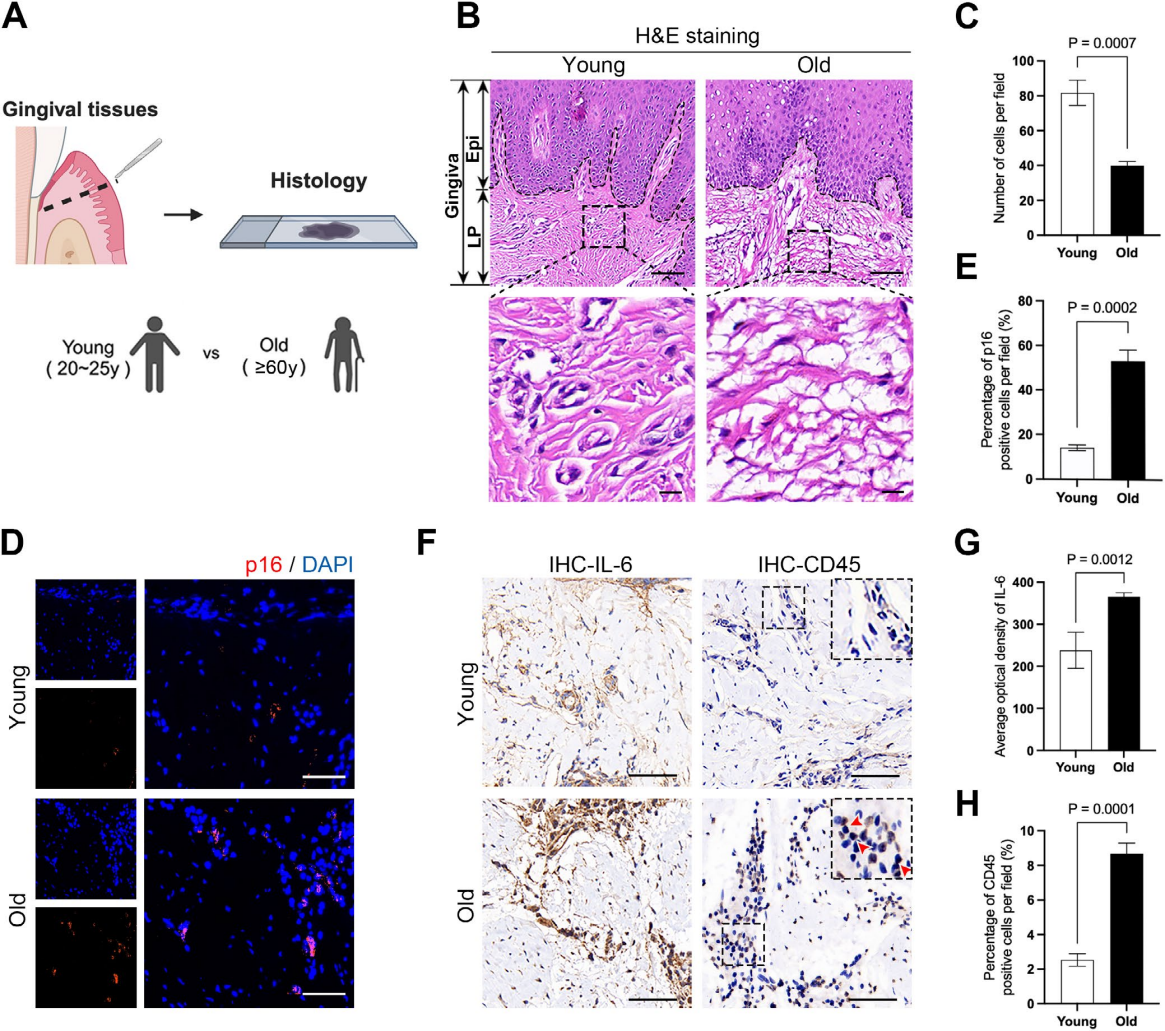
Cellular senescence and inflammation in aging human gingival tissues. (A) Schematic of human gingival tissue test. (B) Representative H&E‐stained images of young and aged gingival tissues. Black dashed lines indicate the boundary between epithelium (Epi) and lamina propria (LP). Scale bars: 100 μm. Insets (bottom panels) show high‐magnification views of the lamina propria. Scale bars: 10 μm. (C) Quantification of fibroblasts numbers in the lamina propria (*n* = 3), showing a decrease in aged gingival tissues (Cohen's *d* = 7.83). (D) Immunofluorescence (IF) images of p16 (senescence marker). Red: P16; blue: DAPI. Scale bars: 100 μm. (E) Quantification of p16‐positive cells (*n* = 3), showing increased senescence in aged tissues (Cohen's *d* = 10.64). (F) Immunohistochemistry (IHC) for IL‐6 and CD45 in gingival tissues. Red arrows indicate CD45‐positive immune cells. Scale bars: 25 μm. Panels (B, F) represent images from adjacent tissue regions rather than identical microscopic fields due to the use of different staining procedures. (G–H) Quantification of IL‐6 and CD45 levels (*n* = 3), showing increased inflammation with aging (Cohen's *d* = 4.05 in IL‐6 and 12.26 in CD45). Unpaired Student's *t*‐tests were used for comparisons between two groups. Data are shown as mean ± SD.

To evaluate the level of cellular senescence of GFs within the aged gingiva, gingival biopsies from young and older individuals were analyzed for histological analysis (Figure 1A). In vivo, H&E staining revealed fewer fibroblasts of the lamina propria in aged gingiva compared to young ones (Figure 1B,C), and immunofluorescence (IF) showed less fibroblasts in the lamina propria of elderly individuals (Figure S1A,B). Masson's staining showed a marked increase in fibrosis in aged gingiva (Figure S1C,D). Stable cell‐cycle arrest has been one of the core features of cellular senescence, and the expression of proteins involved in maintaining proliferative arrest can be used as the primary criterion when assessing senescence. p16Ink4a (CDKN2A locus; encoding p16Ink4a) is one of the inhibitors most commonly expressed by senescent cells. We tested p16 protein levels through immunofluorescence (IF) and found more p16‐positive cells existed in aged gingiva than in young ones (Figure 1D,E), indicating senescent cells accumulated along aging. Moreover, the strength of the senescence‐associated secretory phenotype (SASP), as IL‐6, can vary depending on the duration of senescence and is a core feature of cellular senescence (Gorgoulis et al. 2019). CD45, or leukocyte common antigen, is exclusively expressed on immune cells (Salehi Farid et al. 2025). Immunohistochemistry (IHC) analysis demonstrated a significant increase in IL‐6 and more CD45‐positive cells in aged gingiva than in young ones (Figure 1F–H), indicating enhanced inflammatory responses in aged gingiva. Besides, cell proliferation can be approximated by measuring the levels of proteins involved in cell cycle progression, such as Ki67, which is recommended as an auxiliary marker to be coupled to the expression level of cell cycle inhibitors (Ogrodnik et al. 2024). Thus, the percentage of Ki67‐positive cells was decreased in aged gingiva, further supporting the accumulation of senescent cells (Figure S1E,F). These findings suggest that cellular senescence and inflammation are increased in aged human gingiva.

### 2.2 scRNA‐Seq Indicated Variation of Cilia‐Related Genes Expression in Gingival Fibroblasts Along Aging

**FIGURE 2:**
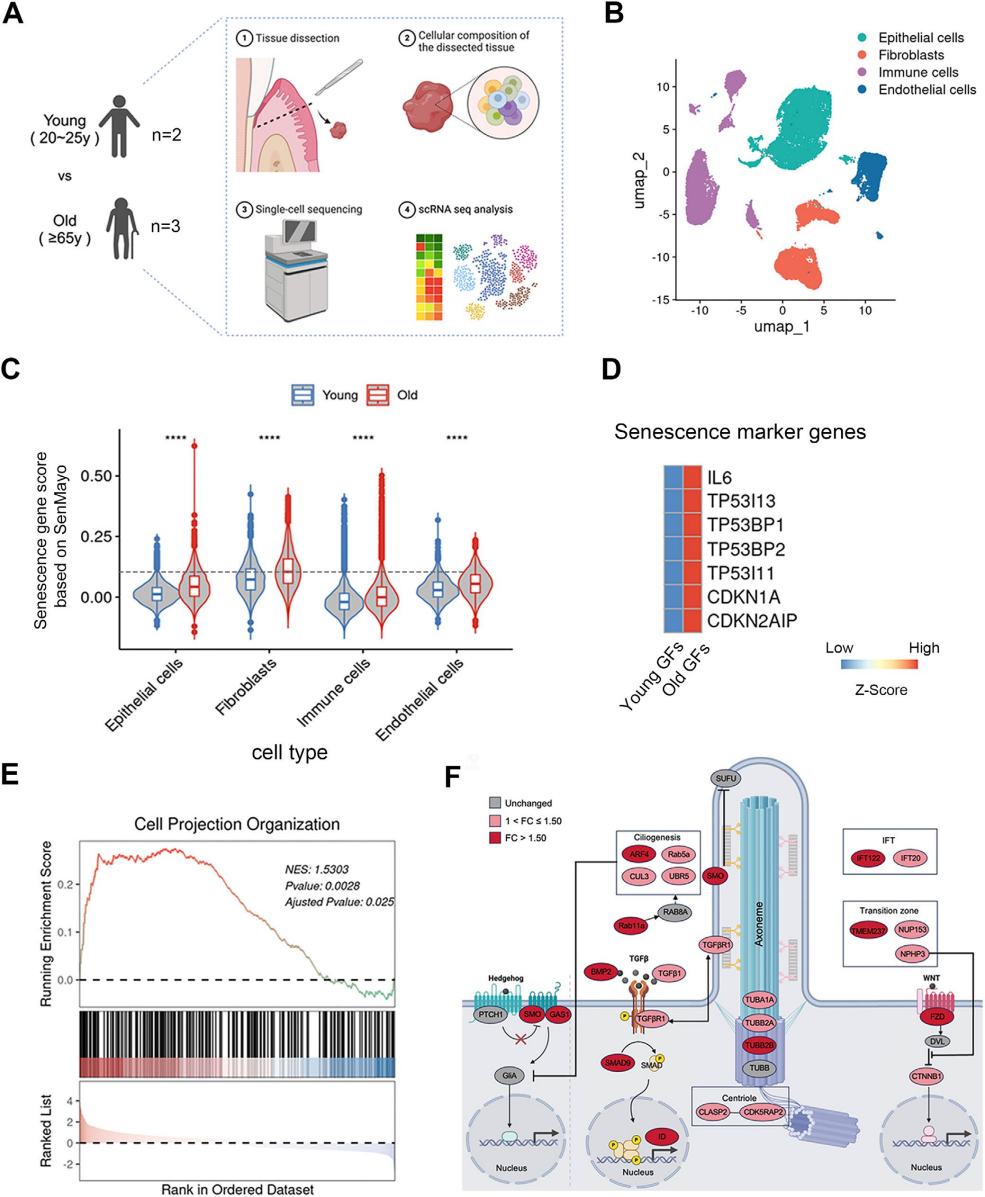
Enhanced cellular senescence and altered cilia‐related gene expression in aging gingival fibroblasts. (A) Workflow of scRNA‐seq analysis comparing young and aged gingival tissues (*n* = 2, young; *n* = 3, aged). (B) UMAP plots showing cell type distribution in gingival tissues. (C) Comparison of SenMayo senescence scores across major cell types. Fibroblasts in aged tissues showed the highest increase (1.47‐fold). (D) Heatmap of senescence markers, most of which are upregulated in aged GFs. (E) GSEA showing enrichment of genes related to cell projection organization, including cilia and axons, in aged GFs. (F) Visualization of upregulated cilia‐associated DEGs in aged GFs. Color intensity reflects fold change (FC).

To investigate gingival aging at the molecular level, we performed single‐cell RNA sequencing (scRNA‐seq) on healthy gingival tissues from young individuals (aged 20–25 years) and elderly individuals (aged over 65 years), grouped as two young and three old cohorts (Figure 2A). Sixteen distinct cellular clusters with their top genes were identified via unbiased clustering combined with uniform manifold approximation and projection (UMAP) analysis (Figure S2A). And then, these clusters were grouped into four major cell types: epithelial cells, fibroblasts, immune cells, and endothelial cells (Figure 2B, Figure S2B). The classification accuracy of these cell types was further validated with additional markers specific to each population (Figure S2C). SenMayo gene set, which includes 125 genes, has been validated for accurately characterizing senescent cells at the single‐cell level (Saul et al. 2022). Based on SenMayo scores, obvious increase was observed across all kind of cells in the gingival tissue of the elderly group compared to the young group, whereas fibroblasts displayed the most pronounced upregulation (Figure 2C). Notably, senescence‐related genes including CDKN2A1P, CDKN1A, TP53I11, and IL6 were significantly elevated in elderly GFs (Figure 2D), indicating proliferation arrest and increased secretion of the SASP in fibroblasts from aged gingival tissue.

Gene Ontology (GO) enrichment based on the top 500 differentially expressed genes (DEGs) between elderly and young GFs revealed upregulation of biological processes related to cell proliferation, senescence, TGF‐β and FGF signaling, and endoplasmic reticulum stress. Besides that, the functions of fibroblast including extracellular matrix synthesis and inflammatory responses were markedly altered in elderly GFs. Notably, the cytoskeleton—particularly the microtubule network organization—also underwent changes (Figure S2D). Cilia, as specialized microtubule‐based structures, function in sensing and transducing extracellular signals. Consistently, Kyoto Encyclopedia of Genes and Genomes (KEGG) pathway enrichment analysis revealed changes in aging‐related pathways, including the p53, FoxO, and TGF‐β signaling pathways, whereas also uncovering changes in cilia‐associated signaling pathways, such as Wnt and TGF‐β signaling (Figure S2E). Gene Set Enrichment Analysis (GSEA) further highlighted activation of cell projection organization, specifically for cilia assembly, in elderly fibroblasts (Figure 2E). These findings inspired us to investigate the changes in cilia within GFs during aging.

Based on previously reported cilia‐associated gene sets (Chen et al. 2021; van Dam et al. 2013, 2019), we identified significant upregulation of cilia assembly genes, including Intraflagellar Transport genes (IFT20 and IFT122), axoneme‐related genes (TUBA1A, TUBB2A, and TUBB2B), transition zone genes (TMEM237, NUP153, and NPHP3), and centrosome genes (CLASP2 and CDK5RAP2). Additionally, non‐structural genes involved in ciliogenesis, such as ARF4, Rab5a, Rab11, CUL3, and UBR5, were also markedly upregulated (Figure 2F). These findings prompted further investigation into the role of primary cilia in gingival fibroblast aging.

### 2.3 Aged Human Gingival Fibroblasts Exhibit Significant Cellular Senescence

**FIGURE 3:**
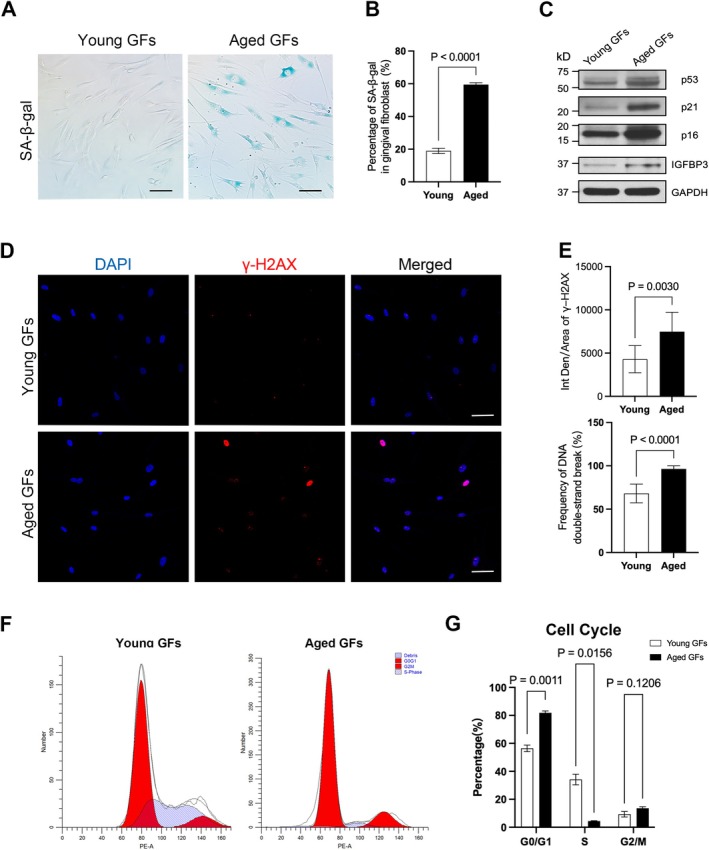
Cellular senescence in aged human gingival fibroblasts. (A) SA‐β‐gal staining of young and aged GFs. Blue‐stained cells indicate senescence. Scale bars: 100 μm. (B) Quantification of SA‐β‐gal‐positive cells (*n* = 3), showing increased senescence in aged GFs. (C) Western blot and quantification of p53, p21, p16, and IGFBP3 expression in young and aged GFs (*n* = 3). Senescence markers were upregulated in aged GFs. (D) IF of γ‐H2AX (DNA damage marker). Red: γ‐H2AX; blue: DAPI. Scale bars: 100 μm. (E) Quantification of γ‐H2AX intensity and DNA damage frequency (*n* = 3), showing increased DNA damage in aged GFs. (F–G) Flow cytometry analysis of cell cycle phases in young and aged GFs (*n* = 3). Aged GFs showed cell cycle arrest (G0/G1 phase). Unpaired Student's *t*‐tests were used for comparisons between two groups. Data are shown as mean ± SD.

To further examine the senescent signatures of aged GFs, we isolated primary GFs from healthy human gingiva across an age range of 18 to 75 years (Table S1) and cultured them in vitro. One of the most recognizable and common markers of cellular senescence is senescence‐associated beta‐galactosidase (SA‐β‐gal), which reflects the activity of a lysosomal enzyme that cleaves terminal β‐d‐galactose residues from β‐D‐galactosides (Ogrodnik et al. 2024). Similarly, in vitro, the percentage of SA‐β‐gal‐positive cells increased significantly, from 18.95% ± 1.64% in young GFs to 59.48% ± 1.17% in aged GFs, confirming the vulnerability of GFs to aging (Figure 3A,B). The protein level of p53, p21, p16, classic senescence markers, and IGFBP3, the SASP marker, were upregulated in aged GFs as well (Figure 3C, Figure S3A). γ‐H2AX was a key indicator of DNA double‐strand breaks (DSBs), one direct cause leading to senescence (Ogrodnik et al. 2024). Aged GFs exhibited increased fluorescence intensity of γ‐H2AX and a higher proportion of γ‐H2AX‐positive cells, indicating elevated DNA damage (Figure 3D,E). Cell cycle arrest leads to a reduction in cell proliferation, which is regarded as a core marker of cellular senescence (Ogrodnik et al. 2024). We detected the cell cycle distribution through flow cytometry analysis. The results revealed that the proportion of cells arrested in the G0/G1 phase increased by 1.45‐fold in aged GFs compared to young GFs, providing further evidence of growth arrest in aged GFs (Figure 3F,G). Taken together, both aged human gingiva and primary GFs from aged individuals exhibited several characteristics of senescence.

### 2.4 Suppression of Ciliogenesis Reverses Gingival Fibroblast Senescence

**FIGURE 4:**
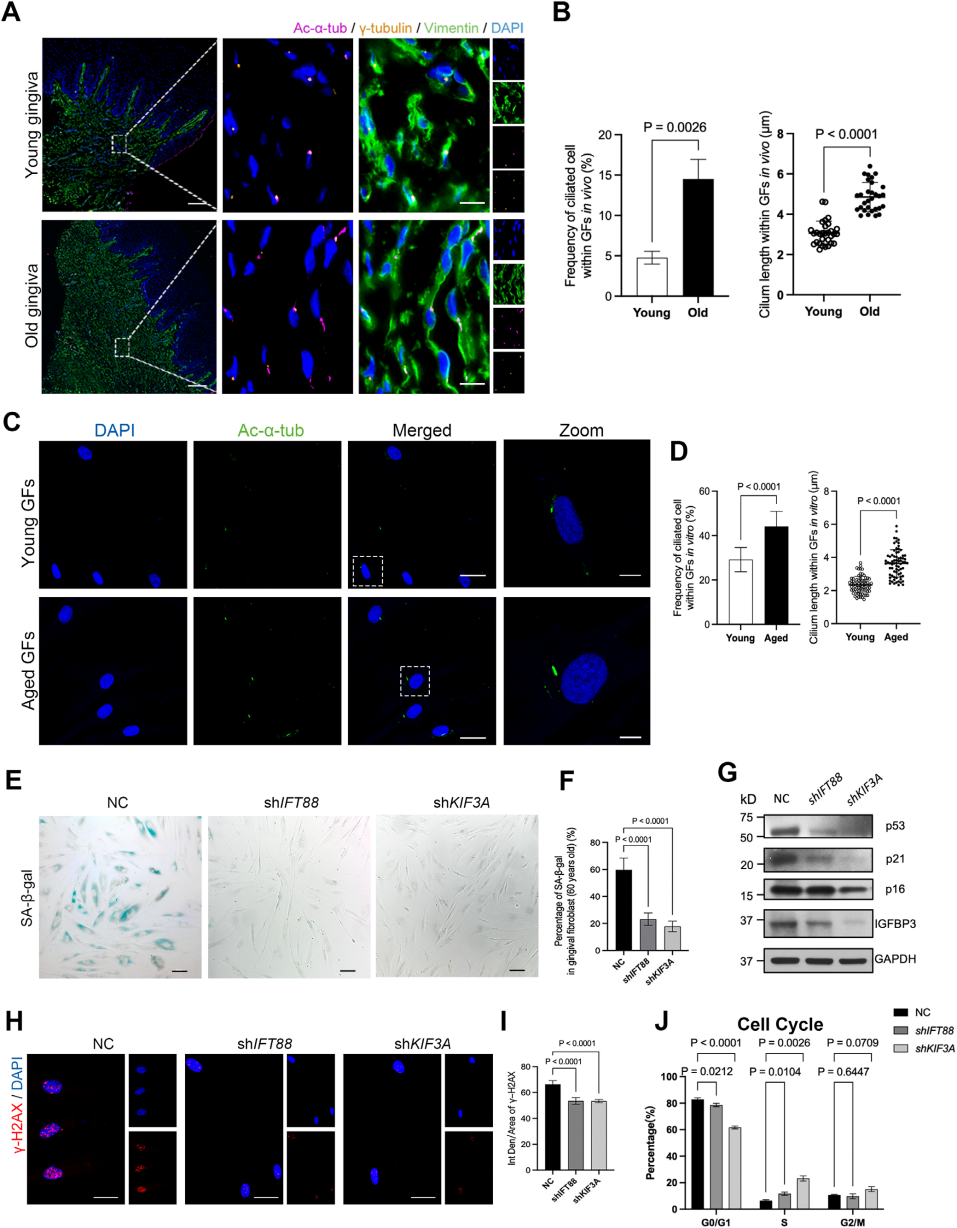
Suppression of ciliogenesis reduces senescence in aged gingival fibroblasts. (A) F images of primary cilia in young and aged gingival tissues. Pink: Ac‐α‐tubulin; yellow: γ‐tubulin; green: Vimentin; blue: DAPI. Scale bars: 100 μm. Insets (right panels) show high‐magnification views of cilia. Scale bars: 10 μm. (B) Quantification of ciliation frequency (*n* > 100) and ciliary length (*n* > 30) in gingival tissues. Both increased in aged tissues (Cohen's *d* = 5.67 for ciliation frequency and 2.81 for ciliary length). (C, D) IF images of primary cilia and quantification in serum‐starved young and aged GFs (*n* > 300 for frequency; *n* > 70 for length). Scale bars: 50 μm. Insets (right panels) show high‐magnification views of individual cilia. Scale bars: 12.5 μm. (E) SA‐β‐gal staining of senescent cells in negative control (NC), shIFT88‐, and shKIF3A‐treated aged GFs at 7 days post‐shRNA viral infection. Scale bars: 100 μm. (F) Quantification of SA‐β‐gal‐positive cells (*n* = 3). Senescent cells decreased following cilia inhibition (Cohen's *d* = 5.29 (NC vs. shIFT88), 7.25 (NC vs. shKIF3A)). (G) Western blot analysis of p53, p21, p16, and IGFBP3 protein levels in NC, shIFT88‐, and shKIF3A‐treated GFs. Senescence markers decreased after cilia inhibition. (H) IF images of γ‐H2AX (DNA damage marker) in NC, shIFT88‐, and shKIF3A‐treated aged GFs. Red: γ‐H2AX; blue: DAPI. Scale bars: 25 μm. (I) Quantification of γ‐H2AX fluorescence intensity (*n* = 3). DNA damage decreased following cilia inhibition (Cohen's *d* = 4.56 (NC vs. shIFT88) and 6.56 (NC vs. shKIF3A)). (J) Flow cytometry of cell cycle phases in NC, shIFT88‐, and shKIF3A‐treated GFs (*n* = 3). Reduced cell cycle arrest was observed after cilia inhibition. Unpaired Student's *t*‐tests were used for comparisons between two groups, whereas one‐way ANOVA with Bonferroni's multiple comparisons test was applied for comparisons among multiple groups. Data are mean ± SD (*n* = 3).

To investigate the role of primary cilia in the context of GF senescence, we performed IF staining of acetylated α‐tubulin (Ac‐α‐tub), a widely used marker for cilia. The proportion of ciliated GFs increased from 4.77% ± 0.78% in young gingival tissues to 14.52% ± 2.42% in aged tissues, with ciliary length extending from an average of 3.07 ± 0.59 μm in young samples to 4.86 ± 0.72 μm in aged samples (Figure 4A,B). Ciliogenesis in vitro can be stimulated by serum starvation (Tang et al. 2013). Compared to the young GFs, we found a substantial increase in cilia formation and cilia length in primary cultured aged GFs with serum starvation (Figure 4C,D). To explore the relationship between cilia formation and cellular senescence, we compared markers of senescence in ciliated and non‐ciliated cells. The proportion of cells with DSBs was significantly higher in ciliated cells, showing a strong positive correlation with ciliation frequency (*r* = 0.7041, Figure S4A–C). Similarly, the percentage of SA‐β‐gal‐positive cells correlated strongly with the frequency of ciliation (*r* = 0.9239, Figure S4D). These results indicate that cilia formation correlates with cellular senescence.

To further investigate the role of primary cilia in cellular senescence, we knocked down IFT88 and KIF3A, two essential genes for ciliogenesis, in aged human GFs (Figure S5A) (Rosenbaum and Witman 2002). Knockdown of either IFT88 or KIF3A gene significantly reduced both the percentage of ciliated cells and the average cilium length, confirming the effective suppression of ciliogenesis (Figure S5B,C), which refers to genetic impairment of IFT‐dependent primary cilium formation and maintenance. Notably, the percentage of SA‐β‐gal‐positive cells decreased markedly, from 59.76% ± 8.68% in the control group (NC) to 23.14% ± 4.59% and 17.86% ± 3.91% in the shIFT88‐ and shKIF3A‐treated cells, respectively (Figure 4E,F). Immunoblot analysis revealed a substantial reduction in the expression of p53, p21, p16, and IGFBP3 in shIFT88‐ and shKIF3A‐treated aged GFs (Figure 4G, Figure S5D). IF staining further showed that knockdown of IFT88 or KIF3A alleviated intensity of γ‐H2AX in aged GFs (Figure 4H,I). Similarly, the intensity of p53 also decreased in IFT88‐ and KIF3A‐depleted cells (Figure S5E,F). Additionally, mRNA levels of SASP factors, including IL‐6, IL‐1β, and TGF‐β, were significantly decreased in the treated cells (Figure S5G). Regarding the cell cycle analysis, compared to NC, the proportion of cells in the G0/G1 phase decreased 4.25% ± 0.96% and 21.21% ± 0.87% in IFT88‐ and KIF3A‐depleted aged GFs, respectively (Figure 4J, Figure S5H). Collectively, these findings indicate that suppression of primary ciliogenesis alleviates cellular senescence in aged human GFs, highlighting the potential regulatory role of primary cilia in cellular senescence.

### 2.5 Knockdown of IFT88 Reverses Cellular Senescence of Gingival Fibroblasts by Activating the AKT Signaling Pathway

**FIGURE 5:**
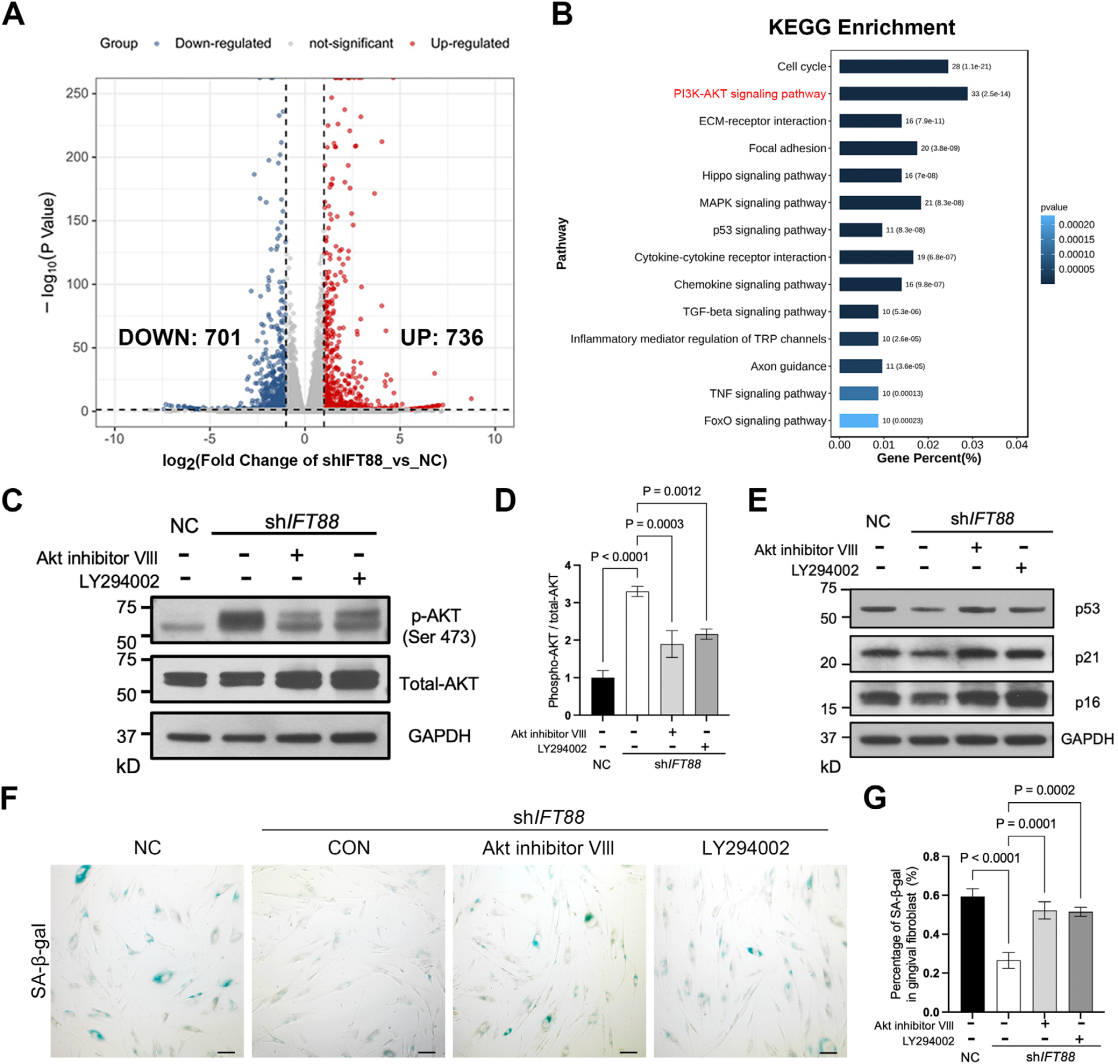
Activation of the AKT signaling in shIFT88‐treated aged gingival fibroblasts. (A) Volcano plot of DEGs between NC and shIFT88‐treated GFs. Thresholds: |log_2_FC| > 1 (vertical dashed line) and *p* < 0.05 (horizontal dashed line). (B) KEGG pathway analysis of DEGs, highlighting activation of the PI3K–AKT pathway in shIFT88‐treated GFs. (C, D) Western blot analysis of AKT phosphorylation (p‐AKT) in NC and shIFT88‐treated GFs, with or without AKT inhibitors VIII (5 μM) or LY294002 (10 μM) treatment for 48 h. p‐AKT levels increased after cilia inhibition and decreased following inhibitor treatment. (E) Western blot analysis of senescence markers (p53, p21, p16) in NC and shIFT88‐treated GFs, with or without AKT inhibitors. Senescence markers decreased with cilia inhibition and increased after AKT/PI3K inhibitor treatment. (F) SA‐β‐gal staining of NC and shIFT88‐treated GFs, with or without AKT inhibitors. Scale bars: 100 μm. (G) Quantification of SA‐β‐gal‐positive cells (*n* = 3). Senescent cells increased following AKT inhibition. Statistical differences were analyzed using one‐way ANOVA with Bonferroni's multiple comparisons test. Data are mean ± SD (*n* = 3).

To further assess how primary cilia ablation impacts senescence phenotypes in aged GFs, bulk‐RNA sequencing analysis of NC and shIFT88‐treated aged GFs was conducted. 1437 DEGs were identified including 736 upregulated and 701 downregulated in shIFT88‐treated GFs (Figure 5A). KEGG pathway analysis revealed enrichment of aging‐related pathways, including the cell cycle, p53, and PI3K–AKT signaling pathways (Figure 5B). GO analysis of the upregulated genes further indicated activation of similar signaling pathways (Figure S6A). Among these, AKT‐related signaling was prominently enriched, suggesting a potential association with ciliary dynamics. Based on these observations, we next examined whether AKT activation is involved in the anti‐senescent effects associated with ciliogenesis inhibition. Indeed, we observed increased levels of phosphorylated AKT in shIFT88‐treated GFs (Figure 5C,D). Furthermore, the PI3K inhibitor LY294002 and the AKT1/2 inhibitor AKT inhibitor VIII significantly suppressed AKT activity and increased p53, p21, and p16 in shIFT88‐treated GFs (Figure 5C–E, Figure S6B). Additionally, inhibition of AKT pathway increased the proportion of SA‐β‐gal‐positive cells, compared to untreated controls (Figure 5F,G). γ‐H2AX staining further showed that AKT inhibition reduced the DNA‐repair benefit conferred by IFT88 knockdown (Figure S6C,D). Together, these results support that AKT activity is functionally associated with the anti‐senescent effects induced by IFT88 knockdown.

To further validate these findings using an alternative approach, we pharmacologically inhibited ciliogenesis in aged GFs using Ciliobrevin D (CD). Immunofluorescence staining confirmed that CD treatment significantly reduced primary cilia frequency in aged GFs (Figure S7A,B). Consistent with the genetic knockdown results, SA‐β‐gal staining demonstrated that CD treatment reduced the proportion of senescent cells, whereas subsequent treatment with AKT inhibitors partially restored senescence‐associated phenotypes (Figure S7C,D). In parallel, qRT‐PCR analysis showed decreased expression of senescence‐associated markers, including TP53, CDKN1A, and CDKN2A, following ciliogenesis inhibition, whereas AKT inhibition partially reversed these effects (Figure S7E). These findings further support a functional role of AKT signaling downstream of ciliary modulation in regulating senescence‐associated phenotypes.

Relevant study has also reported the role of the AKT signaling in nucleus pulposus‐derived mesenchymal stem cell senescence and DNA repair (Huang et al. 2023). FOXO1 is a key transcription factor for DNA repair and is tightly regulated by the AKT signaling, in which AKT negatively regulates the activity of FOXO1 (Lam et al. 2013; Saline et al. 2019). These findings suggest a potential link between AKT signaling and FOXO1 regulation in gingival fibroblasts. We found FOXO1 significantly accumulated in the nuclei of gingival fibroblasts in aged individuals (Figure S6E,F). Consistently, scRNA‐seq also revealed the activation of the FOXO pathway in aged GFs (Figure S2E). These results indicate that FOXO1 might play a key effect between cilia‐mediated AKT pathway and cellular senescence. We next examined FOXO1 phosphorylation and DNA repair‐related genes following pharmacological inhibition of ciliogenesis. Western blot analysis demonstrated that CD treatment increased both AKT phosphorylation and FOXO1 phosphorylation, whereas AKT inhibition attenuated FOXO1 phosphorylation (Figure S7F,G), suggesting that ciliogenesis inhibition may regulate FOXO1 activity through AKT signaling. Moreover, qRT‐PCR analysis revealed decreased expression of the FOXO1 downstream target gene GADD45 after CD treatment, accompanied by increased expression of DNA repair‐related genes, including XRCC5, BRCA1, and RAD51 (Figure S7H). These changes were partially reversed following AKT inhibition. Collectively, these findings further support a functional association between ciliary dynamics, AKT signaling, FOXO1 regulation, and senescence‐associated phenotypes in aged gingival fibroblasts.

### 2.6 Suppression of Ciliogenesis via AAV‐Sh‐IFT88 Mitigates Gingival Aging and Periodontal Inflammation of Mice

**FIGURE 6:**
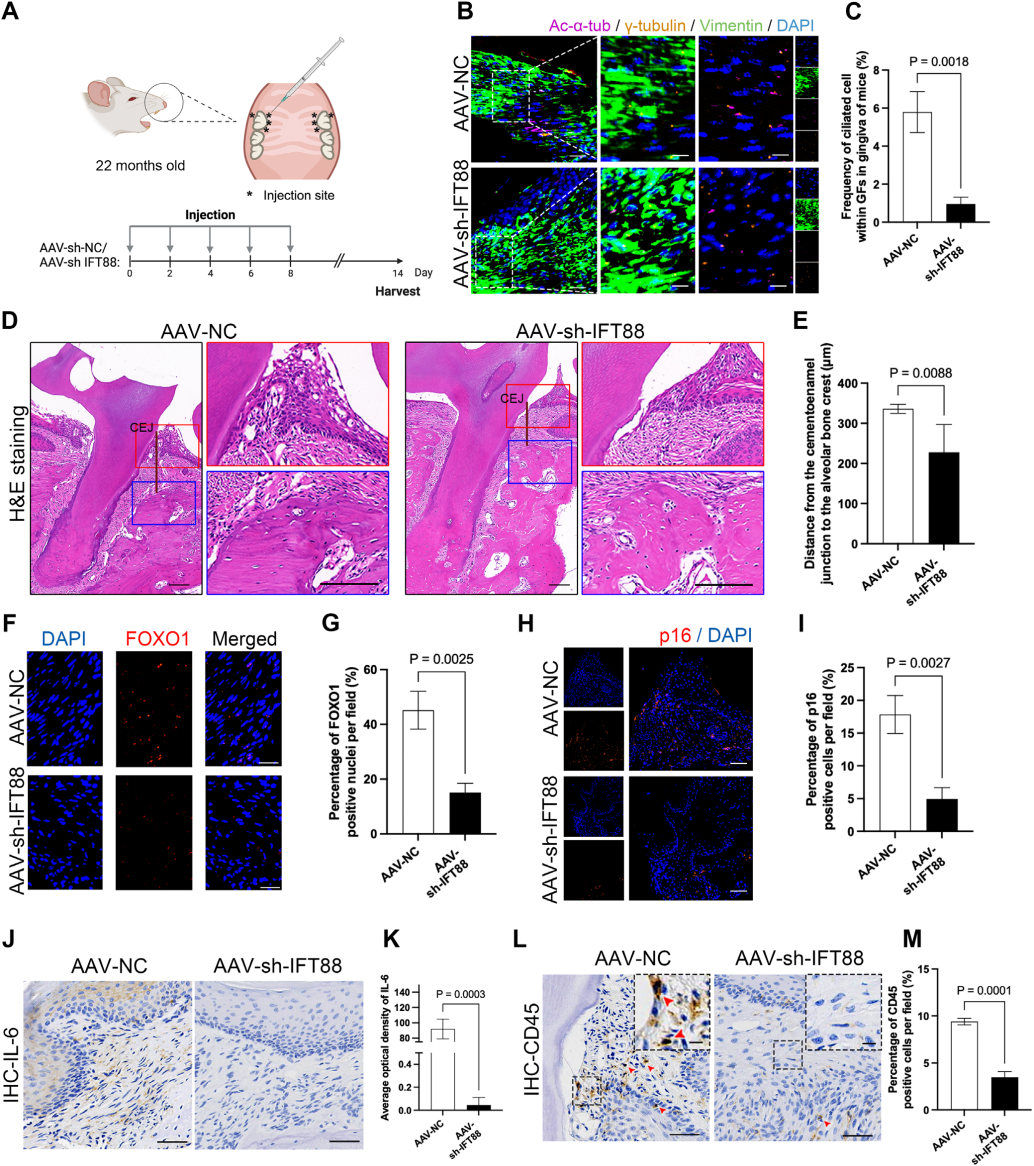
AAV‐shIFT88 injections alleviate gingival aging and inflammation in aged mice. (A) Schematic of AAV injection. (B) IF images of primary cilia in gingival tissues. Pink: Ac‐α‐tub; yellow: γ‐tubulin; green: Vimentin; blue: DAPI. Scale bars: 50 μm. Insets (right panels) show high‐magnification views of primary cilia. Scale bars: 10 μm. (C) Quantification of ciliated cells (*n* = 3). Ciliation decreased after AAV‐shIFT88 injection (Cohen's *d* = 6.18). (D) Representative H&E staining images of the maxilla from mice. The brown lines show the distance from the cementoenamel junction to the alveolar bone crest (CEJ–ABC distance). Regions of interest were identified within a gingival tissue region (red) and an osseous region (blue). Scale bars: 100 μm. (E) Quantification of distance from the cementoenamel junction to the alveolar bone crest (*n* = 3, Cohen's *d* = 2.54). (F) IF images of FOXO1 in gingival tissues. Red: FOXO1; blue: DAPI. Scale bars: 25 μm. (G) Quantification of FOXO1‐positive nuclei (*n* = 3). FOXO1 nuclear localization declined after AAV‐shIFT88 injection (Cohen's *d* = 5.50). (H) IF images of p16 in gingival tissues. Red: P16; blue: DAPI. Scale bars: 100 μm. (I) Quantification of p16‐positive cells (*n* = 3). Cell cycle arrest decreased after AAV‐shIFT88 injection (Cohen's *d* = 5.28). (J) Immunohistochemistry for IL‐6 in gingival tissues. Scale bars: 25 μm. (K) Quantification of IL‐6 optical density (*n* = 3). SASP levels declined after AAV‐shIFT88 injection (Cohen's *d* = 9.78). (L) Immunohistochemistry for CD45 in gingival tissues. Red arrows indicate CD45‐positive cells. Scale bars: 25 μm. Insets show high‐magnification views highlighting CD45‐positive cells. Scale bars: 7.5 μm. (M) Quantification of CD45‐positive cells (*n* = 3). Inflammation decreased after AAV‐shIFT88 injection (Cohen's *d* = 12.35). Unpaired Student's *t*‐tests were used for comparisons between two groups. Data are mean ± SD (*n* = 3).

To investigate the role of primary cilia in gingival aging in vivo, adeno‐associated virus (AAV)‐sh‐IFT88 was injected into one side of the maxillary gingiva in 22‐month‐old aged mice, whereas AAV‐sh‐NC was injected into the corresponding location on the opposite side as the negative control (Figure 6A). Based on scRNA‐seq data showing predominant IFT88 expression in GFs, local AAV delivery was expected to primarily target fibroblasts. IF staining of maxillary tissues revealed that the average ciliation rate of GFs decreased from 5.80% ± 1.08% to 1.00% ± 0.36% in the AAV‐sh‐IFT88‐treated side compared to the AAV‐sh‐NC control side (Figure 6B,C), confirming that AAV‐sh‐IFT88 effectively inhibited ciliogenesis in vivo.

Next, we examined whether ciliogenesis inhibition could mitigate gingival aging and periodontal inflammation in mice. First, we measured the distance between the cementoenamel junction and alveolar bone crest (CEJ–ABC) signifying periodontal attachment loss. The increase of CEJ–ABC distance indicates less bone recession after AAV‐sh‐IFT88 injection (Figure 6D,E). The proportion of p16‐positive cells decreased by 72.24% ± 6.16% in the gingiva treated with AAV‐sh‐IFT88 (Figure 6H,I), whereas the percentage of Ki67‐positive cells increased from 9.32% ± 1.45% to 21.50% ± 4.38% (Figure S8A,B). We also assessed the level of IL‐6 and found that the intensity of IL‐6 was nearly undetectable in the AAV‐sh‐IFT88 group (Figure 6J,K), demonstrating that SASP and the inflammatory response were also alleviated. These results indicated that interfering with ciliogenesis alleviated cell cycle arrest, promoted cell proliferation, and mitigated gingival aging in the gingiva of aged mice.

From the perspective of local immune response and inflammation, we also assessed inflammation markers and found that the intensity of IL‐6 was decreased in the AAV‐sh‐IFT88 group, and CD45‐positive cells were scarce (Figure 6J–M). The reduction in CD45 and IL‐6 levels in gingival tissue suggests a mitigation of inflammation in periodontal soft tissues. Furthermore, Masson's staining of gingiva showed a 34.33% ± 3.48% reduction in collagen fiber content in the AAV‐sh‐IFT88‐treated group (Figure S8C,D), indicating diminishing fibrosis. Combined with the periodontal attachment loss, periodontal soft and hard tissues exhibited phenotypes indicative of inflammation resolution after AAV‐sh‐IFT88 injection. In summary, these findings indicate that suppression of ciliogenesis significantly alleviated gingival aging and periodontal inflammation in vivo, possibly via the AKT/FOXO pathway.

## 3 Discussion

In this study, based on single‐cell transcriptomic data, we identified cilia‐associated mechanisms underlying gingival fibroblast senescence in age‐related periodontal disease, and revealed that the AKT signaling acts downstream of ciliary dynamics using complementary in vivo and in vitro models. Compared with reported susceptible epithelial cells as the primary driver of gingival aging (Hu et al. 2024), our findings highlight fibroblast senescence as a critical pathogenic contributor to age‐related periodontal dysfunction. This aligns with recent evidence that senescent fibroblasts promote inflammation and accelerate the progression of periodontitis (Guo et al. 2024), reinforcing the importance of dissecting cell‐type‐specific senescence programs.

Specifically, we found that excessive stabilization of primary cilia contributes to increased DNA damage, cell cycle arrest, and SASP secretion in aged gingival fibroblasts, reinforcing the functional link between ciliary dynamics and senescence. Notably, increased ciliogenesis in aging cells may initially represent an adaptive or compensatory response to cellular stress, whereas excessive or sustained ciliary stabilization may subsequently maintain and reinforce the senescent state. Consistent with these findings, ciliary genes are upregulated in an age‐dependent manner in specific brain tissues (Chen et al. 2021), and their dysfunction impairs mucosal immunity (Dhar et al. 2025). Furthermore, extrinsic stressors such as radiation, oxidative stress, and inflammation could induce ciliogenesis and promote senescence (Ma et al. 2023). However, in models of age‐related diseases such as Alzheimer's disease and amyotrophic lateral sclerosis, significant reductions in ciliogenesis have been observed in specific brain regions and neurons (Chakravarthy et al. 2012; Ma et al. 2011). This apparent divergence highlights the context‐dependent nature of cilia‐senescence interactions, likely reflecting differential cellular reliance on ciliary signaling across tissues and cell types. Importantly, primary cilia are essential sensory organelles that regulate multiple signaling pathways involved in tissue homeostasis, and their dysregulation has been linked to a broad spectrum of disorders. Therefore, indiscriminate or long‐term inhibition of ciliogenesis may carry potential safety risks. In this context, our findings support the concept that transient or context‐dependent modulation of ciliary dynamics, rather than sustained suppression, may represent a more feasible and safer therapeutic strategy.

Intriguingly, our findings demonstrate that suppression of ciliogenesis in aged GFs alleviates senescence phenotypes through increased AKT activation. Previous studies have established that AKT localizes to the basal body of primary cilia (Suizu et al. 2016) and contributes to primary cilia assembly (Zhu et al. 2009). Moreover, suppression of primary cilia has been reported to increase AKT phosphorylation at S473 (Li et al. 2022), a finding consistent with our observations. Mechanistically, analogous to thick actin bundles that sequester signaling molecules (Park et al. 2020), the hyperstable microtubules of elongated cilia may restrict AKT accessibility, a hypothesis that warrants further investigation. Furthermore, the alleviation of GF senescence upon loss of primary cilia was reversed by pharmacological inhibition of AKT activity, supporting a functional role of this pathway in linking cilia dynamics to senescence. Previous studies have shown the role of the AKT signaling pathway in nucleus pulposus‐derived mesenchymal stem cell senescence and DNA damage repair (Huang et al. 2023). With respect to downstream signaling, FOXO1 is a well‐established AKT target involved in stress responses and genomic stability (Lam et al. 2013). In our study, ciliogenesis inhibition was accompanied by FOXO1 inactivation, reduced DNA damage, and increased expression of DNA repair–related genes, suggesting that modulation of FOXO1 activity and DNA repair may reflect downstream adaptations to altered AKT‐dependent stress signaling. Consistently, FOXO1 inactivation has been reported to be associated with altered DNA damage responses in specific biological settings (Sun et al. 2023). Collectively, these findings support a model in which primary cilia regulate senescence‐associated phenotypes through AKT‐dependent signaling, with FOXO1 and DNA repair responses acting as potential downstream effectors.

Currently, the molecular basis of gingival aging and gingival fibroblast senescence is still poorly understood. Our study provides a perspective suggesting that modulation of primary cilia may represent a potential strategy to alleviate senescence‐associated phenotypes and control chronic periodontal inflammation. Mechanistically, knockdown of the ciliary protein IFT88 may alleviate senescence‐associated phenotypes and SASP through AKT signaling. Nevertheless, this study has several limitations. First, the factors driving excessive primary cilia stabilization during gingival aging remain to be identified. Second, the direct molecular interplay between primary cilia and the AKT signaling was not fully elucidated. Third, the relatively small sample size in the animal experiments may limit the generalizability of the in vivo findings. Fourth, as pooled samples were used for single‐cell analysis, donor‐level heterogeneity may be partially obscured and batch effects cannot be fully excluded. Fifth, both IFT88 and KIF3A have reported roles beyond ciliogenesis. Although two independent knockdown strategies together with a ciliary inhibitor yielded consistent phenotypes supporting a cilia‐associated mechanism, contributions from cilia‐independent functions cannot be fully excluded. In addition, although bilateral intra‐animal injection was used, potential AAV spillover cannot be fully excluded; however, the lack of contralateral effects and consistency with in vitro findings support a predominantly local mechanism. Finally, cilia‐targeted therapies have shown promising results in the treatment of various diseases (Fliegauf et al. 2007; Wang et al. 2025), and how to achieve precise, context‐dependent, and tissue‐specific modulation of ciliary dynamics for oral therapeutic applications remains an important challenge.

In conclusion, this study provides a comprehensive understanding of the relationship between gingival aging and primary cilia. It highlights novel mechanisms underlying aging and proposes tissue‐specific strategies targeting cilia to combat aging‐related periodontal diseases, ultimately aiming to improve periodontal health in the elderly.

## 4 Materials and Methods

### 4.1 Donor Recruitment and Ethical Approval

This study was approved by the Ethics Committee of the Hospital of Stomatology, Wuhan University (WDKQ2025A27) and conducted in accordance with the revised Declaration of Helsinki. This study has complied with the STROBE protocol. Written informed consent was obtained from all participants. All donors were systemically healthy based on a comprehensive medical history review. The inclusion criteria were as follows: (1) good general health without systemic diseases; (2) gingival tissue without erythema, edema, bleeding, and other symptoms; (3) no use of nicotine‐related products in the past 6 months. The exclusion criteria were as follows: (1) patients diagnosed with periodontitis; (2) clinical attachment loss ≥ 3 mm; (3) patients with periodontal pocket. Gingival tissue samples were collected under local anesthesia. Patients aged ≤ 25 years were assigned to the young group, whereas those aged ≥ 60 years were included in the old group. Demographic data, including age and sex, are summarized in Table S1.

### 4.2 Cell Culture

Human gingival fibroblasts were isolated from healthy donors aged 18 to 75. Gingival tissues were aseptically washed in PBS containing 2% penicillin, streptomycin, and amphotericin B. The tissues were cut into 1–2 mm^2^ fragments after removing blood vessels and digested at 37°C for 1 h in DMEM (Gibco) containing 0.5 mg/mL collagenase II (Biofroxx). The fragments were centrifuged at 1000 rpm for 5 min and evenly distributed in a 25 cm^2^ culture dish without liquid for 5 h. Complete DMEM supplemented with 20% fetal bovine serum (FBS; Gibco), 2% penicillin–streptomycin, and amphotericin B was added, and cells were cultured at 37°C in a humidified atmosphere of 5% CO_2_. Medium was replaced every 3 days until cells reached 80% confluence. GFs were harvested and passaged for amplification, with passages 2–10 used in experiments.

### 4.3 Gingival Tissue Dissociation and scRNA‐Seq Library Construction

Clinically collected gingival tissues were dissociated into single‐cell suspensions. To ensure sufficient cell numbers for scRNA‐seq, gingiva from similar donors were pooled as one sample. Cell suspensions were counted with a hemocytometer and diluted to 700–1200 cells/μL. scRNA‐seq libraries were prepared using the Chromium Next GEM Single Cell 3′ Reagent Kits v3.1 (cat. no. 10000268; 10X Genomics), following the manufacturer's instructions. Libraries were sequenced on the BGI DNBSEQ‐T7 platform to a depth of approximately 50,000 reads per cell. The RNA‐seq data have been deposited in the NCBI Gene Expression Omnibus (GEO), and the corresponding GEO accession numbers is GSE312885.

### 4.4 scRNA‐Seq Quality Control and Data Analysis

Fastq files were generated and demultiplexed using the Cell Ranger mkfastq pipeline. The Cell Ranger count pipeline was used for alignment, filtering, and UMI counting, with the GRCh38‐2020‐A reference transcriptome. Data were loaded into R Studio (v4.2.2) using the “load10X” function, and samples were merged into a single Seurat object. Filtering was performed to retain cells with > 200 features and < 20% mitochondrial transcripts. Doublets were identified and removed using the “DoubletFinder” package with a 5% expected doublet rate.

Highly variable genes (HVGs) were identified using the “FindVariableGenes” function in Seurat. PCA was performed on HVGs, and results were visualized using UMAP. Marker genes were identified using the “FindAllMarkers” function and classified into four major cell types: epithelial cells (KRT14, KRT5, S100A2), fibroblasts (LUM, COL3A1, COL1A1, CFD), endothelial cells (VWF, PECAM1), and immune cells (CD69, CD52, CXCR4, PTPRC). Marker genes were visualized using the “FeaturePlot” function.

Differentially expressed genes (DEGs) were identified using the “FindMarkers” function in Seurat, with *p* < 0.05 and fold‐change > 1.5 considered significant. Cellular senescence was assessed using the “AddModule” function with the SenMayo gene list. DEGs in fibroblasts were analyzed using DAVID (Sherman et al. 2022) for GO and KEGG pathway enrichment. GSEA was performed using the “ClusterProfiler” package. Cilia assembly and signaling alterations in fibroblasts were evaluated based on reported cilia‐related gene sets (Ma et al. 2022; Moran et al. 2024; Tian et al. 2023).

### 4.5 Histology

Gingival tissues were fixed in 4% paraformaldehyde for 24–48 h, dehydrated in graded ethanol, embedded in paraffin, and sectioned. Sections were deparaffinized, rehydrated, and stained with hematoxylin and eosin (H&E) or Masson's trichrome.

### 4.6 Immunofluorescence Microscopy

Sections underwent antigen retrieval with Tris‐EDTA buffer, blocked with 3% BSA, incubated with primary antibodies overnight at 4°C, and stained with conjugated secondary antibodies at 37°C for 1 h. Nuclei were visualized using DAPI, and slides were mounted with Antifade Mounting Medium (Beyotime). GFs cultured on chamber slides were fixed in 4% paraformaldehyde, permeabilized with 0.1% Triton X‐100, and stained similarly. Confocal microscopy was performed using a Leica TCS SP8 with a 63× oil objective, and images were processed with LAS AF Lite software.

### 4.7 Immunohistochemistry

Sections underwent antigen retrieval, blocking with 3% BSA, incubation with primary antibodies at 4°C, and staining with biotinylated secondary antibodies. Hematoxylin and DAB‐stained images were analyzed using ImageJ Fiji.

### 4.8 Antibodies and Staining Reagents

Primary antibodies included anti‐IFT20 (gift from G. Pazour; 1:2000 for WB), anti‐Ac‐α‐tubulin and anti‐γ‐tubulin (Sigma, T7451 and T6557; 1:1000 for IF), anti‐Ki67 (Abcam, Ab16667; 1:200 for IF), anti‐p16 (Abcam, Ab211542; 1:100 for IF), anti‐FOXO1 (Abways, CY7234; 1:100 for IF), anti‐CD45 (Cell Signaling Technology, 70,257; 1:400 for IHC), anti‐IL‐6 (Bosterbio, BA4339; 1:400 for IHC), anti‐ARL13B (Proteintech, 17711‐1‐AP; 1:1000 for IF), anti‐vimentin (Abclonal, A19607; 1:1000 for IF), anti‐ɣ‐H2AX (Cell Signaling Technology, #2577; 1:1000 for IF), anti‐p53 (Proteintech, 10,442; 1:10,000 for WB), anti‐p21 (Proteintech, 10,355; 1:3000 for WB), anti‐p16 (Proteintech, 10,883; 1:5000 for WB), anti‐IGFBP3 (Proteintech, 10,189; 1:5000 for WB), anti‐IFT88 (Proteintech, 13,967; 1:5000 for WB), anti‐KIF3A (Proteintech, 13,930; 1:1000 for WB), anti‐AKT (Abmart, T55561; 1:1000 for WB), and anti‐pAKT (Abmart, T40067; 1:1000 for WB), anti‐FOXO1 (Abmart, T55376; 1:1000 for WB), and anti‐pFOXO1 (Abmart, PA5293S; 1:1000 for WB). Secondary antibodies included HRP‐conjugated antibodies against mouse or rabbit IgG (Biodragon, BF03001 and BF03008; 1:10,000), Alexa Fluor 488‐ and 555‐labeled secondary antibodies (Invitrogen, A32731 and A32727; 1:1000). Other staining reagents included anti‐GAPDH‐HRP (Biopm, PMK053S; 1:10,000), DAPI (4′,6‐diamidino‐2‐phenylindole) (Beyotime, C1005; 1:1000), and phalloidin (Beyotime, C2201S; 1:100).

### 4.9 SA‐β‐Gal Assays

Senescence‐associated β‐galactosidase (SA‐β‐gal) activity, a hallmark of cell senescence, was detected using the Senescence β‐Galactosidase Staining Kit (Beyotime) according to the manufacturer's instructions. Cells were washed with PBS, fixed at room temperature, and stained overnight with freshly prepared staining solution in the dark at 37°C without CO_2_. Blue‐stained cells were identified as senescent under a light microscope.

### 4.10 Stable Cell Lines

To stably knock down IFT88 and KIF3A expression in human GFs, lentivirus‐mediated shRNA transduction was performed. Lentiviral particles were generated by transfecting 293 T cells with the pLKO.1 plasmid and helper plasmids using Lipofectamine 2000 (Invitrogen). Culture supernatants were harvested at 48 and 72 h, filtered, and used to infect GFs at 80% confluence in 6‐well plates with 8 μg/mL polybrene (Beyotime). Non‐targeting shRNA served as a control. Primer and target sequences are listed in Table S2. Following overnight infection, cells were cultured for 36 h in fresh medium before selection with 2 μg/mL puromycin (Biosharp) every 48 h. Knockdown efficiency was validated by Western blotting.

### 4.11 Western Blotting

Cells were washed with PBS, lysed in RIPA buffer containing protease inhibitors (Beyotime), and ultrasonicated. Lysates were centrifuged (13,000 rpm, 4°C), and protein concentrations were measured using a BCA Protein Assay Kit (Beyotime). Samples were denatured at 95°C for 10 min, separated on SDS‐PAGE gels, and transferred onto NC membranes. After blocking, membranes were incubated overnight with primary antibodies at 4°C, followed by incubation with HRP‐conjugated secondary antibodies for 1 h at room temperature. Signals were visualized using enhanced chemiluminescence (ECL, Millipore) and analyzed with ImageJ software. All Western blot experiments were conducted in triplicate.

### 4.12 RNA Extraction and qRT‐PCR

Total RNA was extracted using TRIzol Reagent (Thermo Fisher Scientific) and reverse‐transcribed into cDNA with qPCR RT Master Mix (ABclonal, RK20429). Real‐time PCR was performed using Universal SYBR Green Fast qPCR Mix (ABclonal, RK21203) and a LightCycler480 Real‐Time PCR System (Roche). Relative gene expression levels were normalized to GAPDH using the ΔΔCt method. Primer sequences are listed in Table S3.

### 4.13 Flow Cytometry

Cell cycle analysis was performed using flow cytometry. GFs were fixed with 70% ethanol at 4°C for 12–24 h, stained with propidium iodide (PI) from a Cell Cycle and Apoptosis Analysis Kit (Beyotime) for 30 min at 37°C in the dark, and analyzed on a CytoFlex flow cytometer (Beckman Coulter). Data were processed using FlowJo software.

### 4.14 RNA Sequencing

Total RNA from human GFs was extracted using TRIzol Reagent (Thermo Fisher Scientific). mRNA was enriched from total RNA using oligo(dT) magnetic beads. cDNA libraries were prepared and sequenced by The Analysis and Testing Center of Institute of Hydrobiology, Chinese Academy of Sciences. Briefly, libraries were pooled according to the target data amount and sequenced on the Illumina NovaSeq 6000 platform. The RNA‐seq data have been deposited in the NCBI Gene Expression Omnibus (GEO), and the corresponding GEO accession number is GSE312885.

### 4.15 Pharmacological Suppression of Ciliogenesis

To pharmacologically suppress ciliogenesis, aged gingival fibroblasts (GFs) were treated with Ciliobrevin D (MedChemExpress, HY‐122632), a dynein ATPase inhibitor that disrupts intraflagellar transport and primary cilium maintenance. Cells were exposed to 25 μM Ciliobrevin D for 48 h, whereas control cells received an equivalent volume of dimethyl sulfoxide (DMSO). Following treatment, cells were subjected to immunofluorescence staining, SA‐β‐gal staining, quantitative real‐time PCR (qRT‐PCR), and western blot analyses to evaluate ciliation frequency, cellular senescence, and AKT‐FOXO1 signaling activity.

### 4.16 AAV Transduction of Mice

Three aged male healthy C57BL/6J mice (22 months old) from Hubei Experimental Animal Research Center were housed in a pathogen‐free, temperature‐ and humidity‐controlled facility under a 12‐h light/dark cycle to minimize potential confounders. The sample size was decided according to reference. All experiments followed the guidelines of the Animal Research Ethics Committee of Wuhan University School of Stomatology and Hospital, China (No. S0792502031), and adhered to ARRIVE guidelines 2.0. Mice were anesthetized by an intraperitoneal injection (i.p.) of 1% pentobarbital sodium (50 mg/kg). AAV‐sh‐IFT88 or AAV‐sh‐NC (1 × 10^11^ packaged genomic particles total) was injected into the buccal and palatal gingival tissues of maxillary molars every 3 days over 7 days using a Hamilton syringe. AAV vectors were locally injected into anatomically separated gingival regions using minimal volumes (3 μL) to limit potential systemic dissemination. Animals were euthanized on Day 14, and gingiva and maxillae were collected for analysis (*n* = 3). All mice were included in the analysis unless they died.

### 4.17 Statistical Analysis

All statistical analyses were performed using GraphPad Prism v9.0 and presented as mean ± SD. Unpaired Student's *t*‐tests were used for comparisons between two groups, whereas one‐way ANOVA with Bonferroni's multiple comparisons test was applied for comparisons among more than two groups. Sample sizes are provided in the figure legends. Statistical significance was defined as *p* < 0.05.

## Author Contributions

Wenjun Shao and Huihui Yang contributed equally to this work. Wenjun Shao contributed to conception, design, data acquisition, analysis, and interpretation, and drafted the manuscript; Huihui Yang, Chenghu Yin, Yunjie Zhang, and Yixing Xu contributed to data curation, formal analysis, and validation; Wakam Chang and Haibin Xia contributed to data acquisition, analysis, and interpretation, and critically revised the manuscript; Min Wang, Liangliang Fu, and Kaiyao Huang contributed to conceptualization, design, project administration, resources, and critically revised the manuscript. All authors gave their final approval and agree to be accountable for all aspects of work.

## Funding

This work was supported by International Cooperation and Exchange of the National Natural Science Foundation of China, 32261160642. National Natural Science Foundation of China, 32571099, 32370816. Wuhan Children's Hospital project, 2023FEBSJJ001. Science and Technology Development Fund, Macau SAR, 0099/2022/AFJ.

## Ethics Statement

Human subjects: All individuals provided written informed consent and this study was supported by the Ethics Committee of Hospital of Stomatology, Wuhan University (WDKQ2025A27). The animal tests in this study adhere to the guidelines established by the Animal Research Ethics Committee of Wuhan University School of Stomatology and Hospital, China. The Ethics Committee approved the Animal Use research with protocol no. S0792502031.

## Conflicts of Interest

The authors declare no conflicts of interest.

## Supporting information


**Appendix S1:** acel70627‐sup‐0001‐AppendixS1.zip.

## Data Availability

The data in this study are available from the corresponding author upon reasonable request.
