# Supplementary material for: Suppression of Ciliogenesis Alleviates Cellular Senescence via AKT Signaling in Gingival Aging
Source: Aging Cell. 2026 Jul 10;25(7):e70627. doi: 10.1111/acel.70627 (PMC13354758; doi:10.1111/acel.70627)

**Suppression of ciliogenesis alleviates cellular senescence via AKT signaling in gingival aging**

Wenjun Shao^1, #^, Huihui Yang^2, #^, Chenghu Yin^1^, Yunjie Zhang^3^, Yixing Xu^1^, Wakam Chang^4^, Haibin Xia^1^, Min Wang^1, 5, *^, Liangliang Fu^1, *^, Kaiyao Huang^3, *^

**Supporting Information**

[**Appendix Table 1.** Subject information 2](#_Toc231254086)

[**Appendix Table 2.** Sequences of shRNA oligos 3](#_Toc231254087)

[**Appendix Table 3.** Primer sequences for real-time PCR 4](#_Toc231254088)

[**Figure S1.** Aging gingival tissues exhibit elevated senescence phentotpyes *in vivo* 5](#_Toc231254089)

[**Figure S2.** Single-cell RNA sequencing of young and aging gingival tissues. 6](#_Toc231254090)

[**Figure S3.** Aging gingival fibroblasts exhibits elevated senescence phentotpyes *in vitro*. 7](#_Toc231254091)

[**Figure S4.** The linear correlation between cilia and DNA double-strand breaks. 8](#_Toc231254092)

[**Figure S5.** Suppression of ciliogenesis improved senescence phentotpyes in aged GFs 9](#_Toc231254093)

[**Figure S6.** Suppression of ciliogenesis through shIFT88 and shKIF3A improved senescence phenotypes through the AKT signaling 11](#_Toc231254094)

[**Figure S7.** Suppressing ciliogenesis through Ciliobrevin D attenuates senescence phenotypes through the AKT signaling 12](#_Toc231254095)

[**Figure S8.** Suppressing ciliogenesis through AAV-sh-IFT88 attenuates gingival aging in mice 13](#_Toc231254096)

[Western Blot Images 14](#_Toc231254097)

**Appendix Table 1.** Subject information

| Sample | Group | Gender | Age(year) | Tooth position | Periodontal condition | Usage | Label |
| --- | --- | --- | --- | --- | --- | --- | --- |
| 1 | Young | Male | 22 | 26 | Healthy | sc-RNA seq analysis | Young1 |
| 2 |  | Male | 21 | 36 | Healthy |  |  |
| 3 |  | Female | 24 | 37 | Healthy |  | Young2 |
| 4 |  | Female | 20 | 15 | Healthy | Histological analysis |  |
| 5 |  | Male | 21 | 16 | Healthy |  |  |
| 6 |  | Female | 24 | 46 | Healthy |  |  |
| 7 |  | Female | 20 | 47 | Healthy | Primary cell culture |  |
| 8 |  | Male | 22 | 36 | Healthy |  |  |
| 9 |  | Male | 22 | 36 | Healthy |  |  |
| 10 |  | Female | 19 | 46 | Healthy |  |  |
| 11 | Old | Female | 77 | 27 | Healthy | sc-RNA seq analysis | Old1 |
| 12 |  | Male | 72 | 25 | Healthy |  | Old2 |
| 13 |  | Male | 76 | 46 | Healthy |  |  |
| 14 |  | Female | 69 | 46 | Healthy |  | Old3 |
| 15 |  | Female | 66 | 16 | Healthy |  |  |
| 16 |  | Female | 64 | 37 | Healthy | Histological analysis |  |
| 17 |  | Female | 60 | 26 | Healthy |  |  |
| 18 |  | Male | 66 | 16 | Healthy |  |  |
| 19 |  | Female | 60 | 27 | Healthy | Primary cell culture |  |
| 20 |  | Male | 61 | 46 | Healthy |  |  |
| 21 |  | Male | 62 | 47 | Healthy |  |  |
| 22 |  | Male | 60 | 36 | Healthy |  |  |

**Appendix Table 2.** Sequences of shRNA oligos

| Gene | 5'-3' | Primer Sequences (5'- 3') |
| --- | --- | --- |
| Human-shIFT88-1 | Forward Primer | CCGGCCAAGTTCCAAGTGTCAATAACTCG  AGTTATTGACACTTGGAACTTGGTTTTTG |
|  | Reverse Primer | AATTCAAAAACCAAGTTCCAAGTGTCAATA  ACTCGAGTTATTGACACTTGGAACTTGG |
| Human-shIFT88-2 | Forward Primer | CCGGCACGGCAGTTACTAGACCTATCTCGA  GATAGGTCTAGTAACTGCCGTGTTTTTG |
|  | Reverse Primer | AATTCAAAAACACGGCAGTTACTAGACCTA  TCTCGAGATAGGTCTAGTAACTGCCGTG |
| Human-shIFT88-3 | Forward Primer | CCGGGCAGTTACATACTTGAGACAACTCGA  GTTGTCTCAAGTATGTAACTGCTTTTTG |
|  | Reverse Primer | AATTCAAAAAGCAGTTACATACTTGAGACA  ACTCGAGTTGTCTCAAGTATGTAACTGC |
| Human-shKIF3A-1 | Forward Primer | CCGGTTCGACTTCAGATGCTTATTACTCGAG  TAATAAGCATCTGAAGTCGAATTTTTG |
|  | Reverse Primer | AATTCAAAAATTCGACTTCAGATGCTTATTA  CTCGAGTAATAAGCATCTGAAGTCGAA |
| Human-shKIF3A-2 | Forward Primer | CCGGCACAAAGGTTAGAGGTTAAAGCTCGA  GCTTTAACCTCTAACCTTTGTGTTTTTG |
|  | Reverse Primer | AATTCAAAAACACAAAGGTTAGAGGTTAAA  GCTCGAGCTTTAACCTCTAACCTTTGTG |
| Human-shKIF3A-3 | Forward Primer | CCGGAGCAAGAACGCTTGGATATTGCTCGAG  CAATATCCAAGCGTTCTTGCTTTTTTG |
|  | Reverse Primer | AATTCAAAAAAGCAAGAACGCTTGGATATTG  CTCGAGCAATATCCAAGCGTTCTTGCT |
| Negative Control | Non-targeting shRNA | CAACAAGATGAAGAGCACCA |
| Mouse-shIFT88 | Target Sequence | GCCCTCAGATAGAAAGACCAA |

**Appendix Table 3.** Primer sequences for real-time PCR

| Gene | 5'-3' | Primer Sequences (5'- 3') |
| --- | --- | --- |
| Human-GAPDH | Forward Primer | AGGTCGGTGTGAACGGATTTG |
|  | Reverse Primer | GGGGTCGTTGATGGCAACA |
| Human-TGF-β1 | Forward Primer | TACCTGAACCCGTGTTGCTCTC |
|  | Reverse Primer | GTTGCTGAGGTATCGCCAGGAA |
| Human-IL-1β | Forward Primer | ATGATGGCTTATTACAGTGGCAA |
|  | Reverse Primer | GTCGGAGATTCGTAGCTGGA |
| Human-IL-6 | Forward Primer | AGACAGCCACTCACCTCTTCAG |
|  | Reverse Primer | TTCTGCCAGTGCCTCTTTGCTG |
| Human-GADD45 | Forward Primer | CTGGAGGAAGTGCTCAGCAAAG |
|  | Reverse Primer | AGAGCCACATCTCTGTCGTCGT |
| Human-BRCA1 | Forward Primer | CTGAAGACTGCTCAGGGCTATC |
|  | Reverse Primer | AGGGTAGCTGTTAGAAGGCTGG |
| Human-RAD51 | Forward Primer | TCTCTGGCAGTGATGTCCTGGA |
|  | Reverse Primer | TAAAGGGCGGTGGCACTGTCTA |
| Human-XRCC5 | Forward Primer | GTTCTAAAGGTCTTTGCAGCAAGA |
|  | Reverse Primer | AAAAGCCACGCCGACTTGAGGA |
| Human-TP53 | Forward Primer | CCTCAGCATCTTATCCGAGTGG |
|  | Reverse Primer | TGGATGGTGGTACAGTCAGAGC |
| Human-CDKN1A | Forward Primer | TTCCACCAGCATGGCAACGTCT |
|  | Reverse Primer | AGCTCCGCGTATTTGCTTTGGG |
| Human-CDKN2A | Forward Primer | CTCCGGAAGCTGTCGACTTC |
|  | Reverse Primer | TTCTGCCATTTGCTAGCAGTGT |


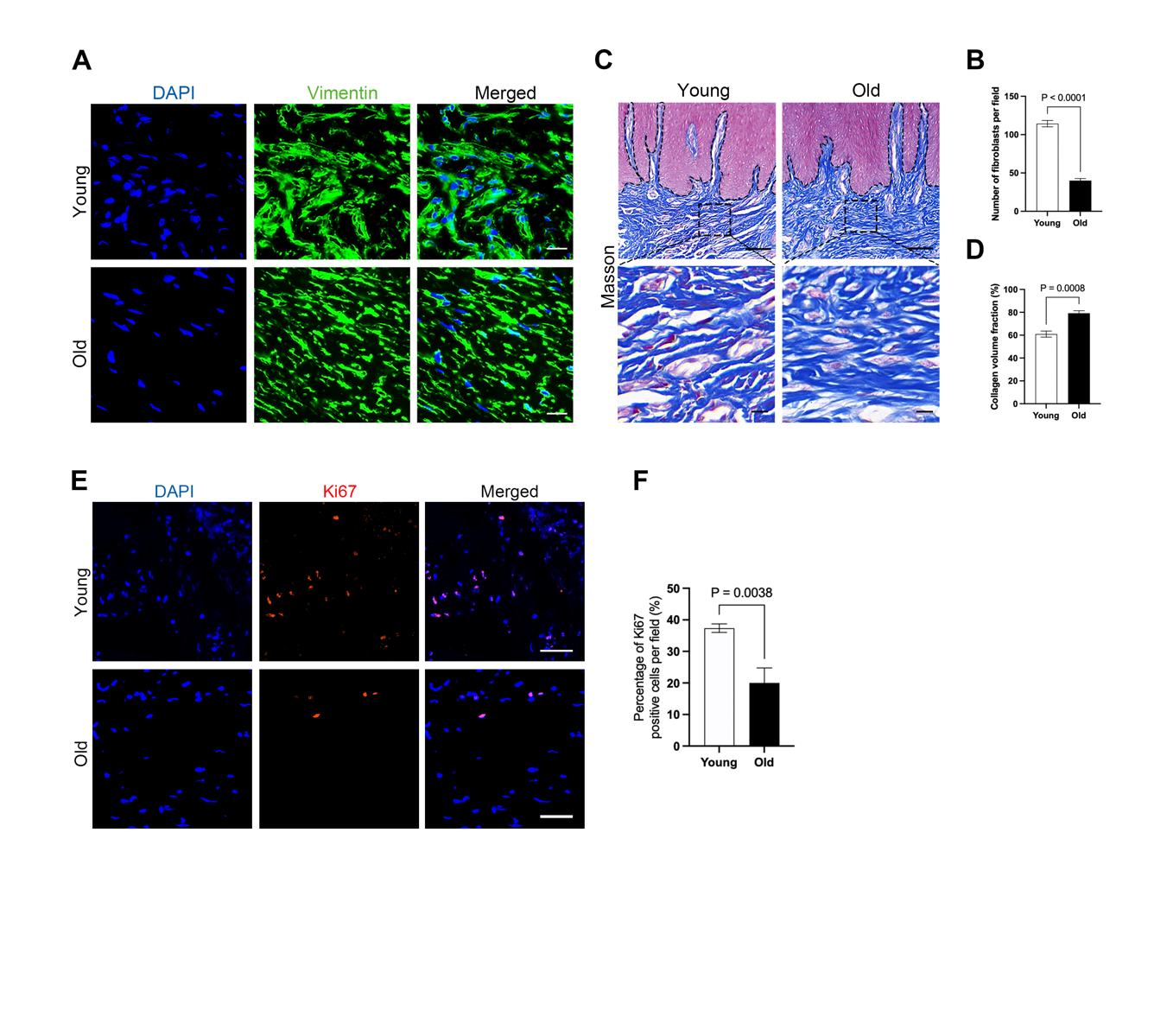


**Figure S1.** Aging gingival tissues exhibit elevated senescence phentotpyes *in vivo*

(A) Immunofluorescence (IF) images of GFs in young and aged gingival tissues. Green: Vimentin (GF marker); blue: DAPI. Scale bars: 20 μm.

(B) Quantification of GFs per field (n = 3). The number of GFs decreased with aging.

(C) Masson’s trichrome-stained images of young and aged gingival tissues. Scale bars: 100 μm. Insets (bottom panels) show high-magnification views of the lamina propria. Scale bars: 10 μm.

(D) Quantification of collagen volume in the lamina propria (n = 3), indicating fibrosis accumulation with aging.

(E) IF images of Ki67 (proliferation marker) in gingival tissues. Red: Ki67; blue: DAPI. Scale bars: 20 μm.

(F) Quantification of Ki67-positive cells (n = 3), showing reduced proliferation with aging.

Statistical differences were analyzed using t-tests. Data are mean ± SD (n = 3).

**
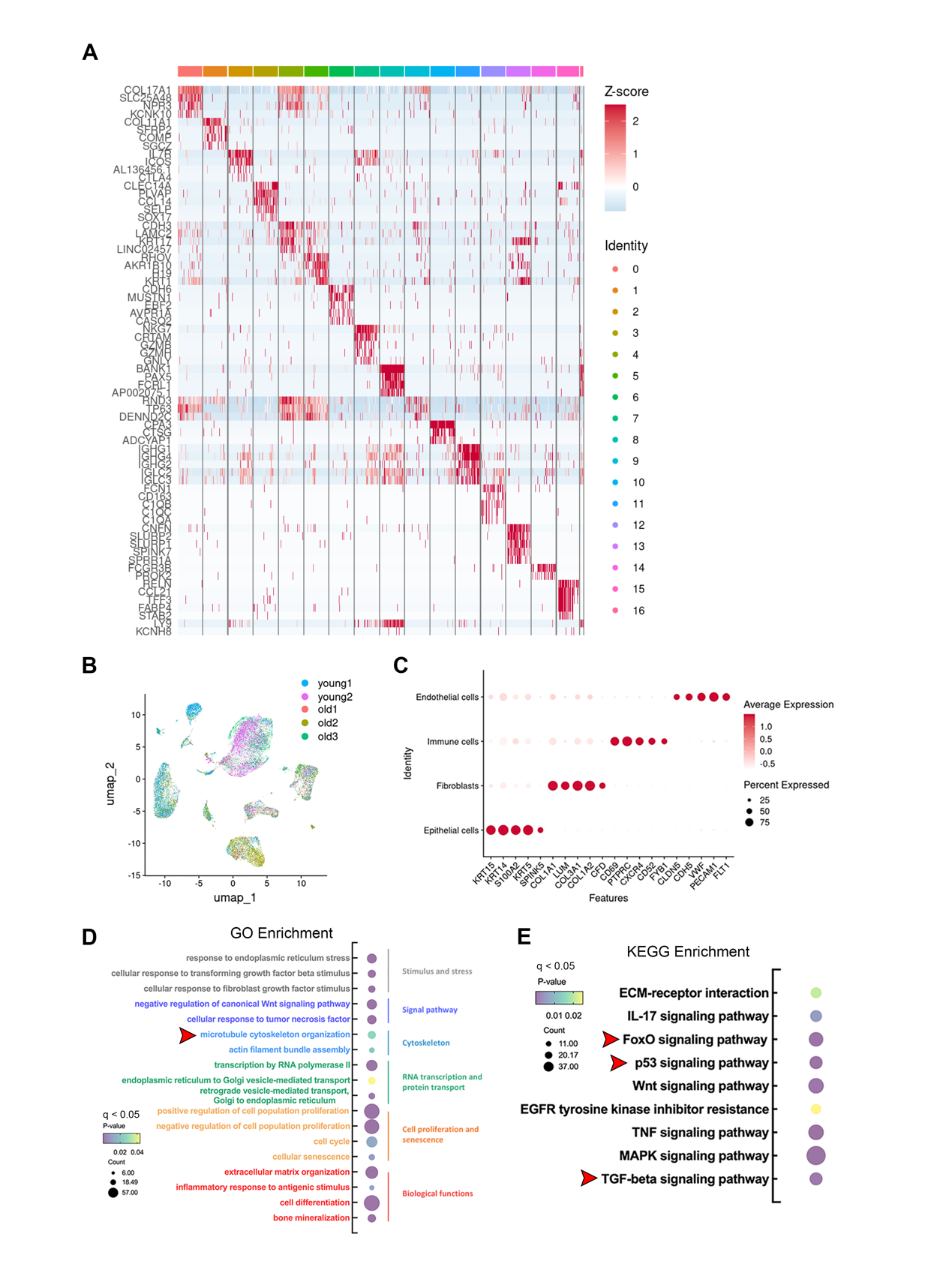
**

**Figure S2.** Single-cell RNA sequencing of young and aging gingival tissues.

(A) Heatmap showing top cell type-specific marker gene expression profiles in different gingival cell types.

(B) UMAP clustering reveals distinct cellular clusters in young and aged tissues.

(C) Dot plots highlighting representative markers for major cell populations and their relative expression in human gingival tissues.

(D) GO enrichment based on upregulated DEGs in aged GFs.

(E) KEGG pathway enrichment based on upregulated DEGs in aged GFs, highlighting aging-related pathways.

**
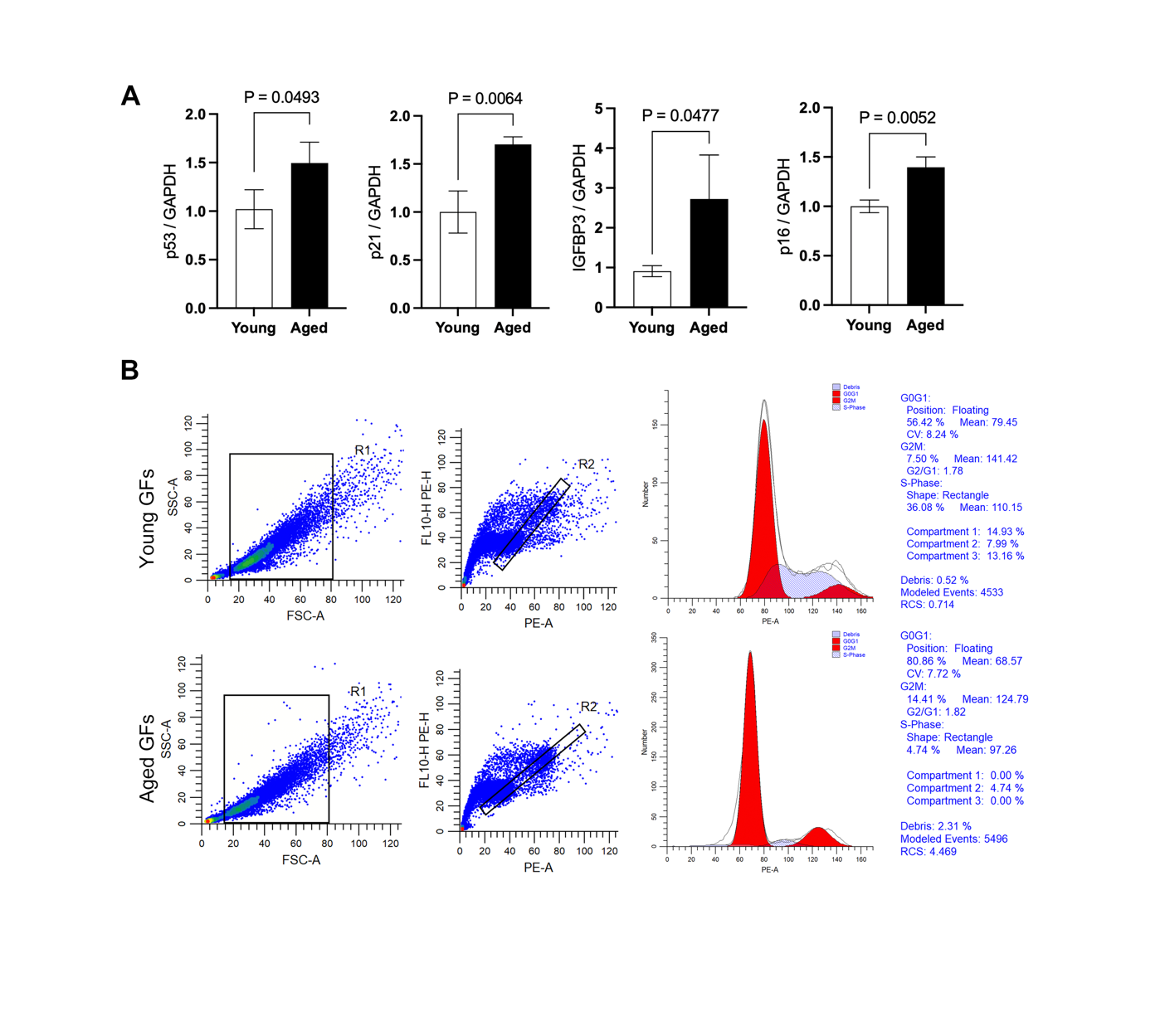
**

**Figure S3.** Aging gingival fibroblasts exhibits elevated senescence phentotpyes *in vitro*.

(A) Quantification of protein expression (n = 3) from Fig. 3C.

(B) Gating strategy of young and aged human GFs flow cytometry.

Statistical differences were analyzed using t-tests. Data are mean ± SD (n = 3).

**
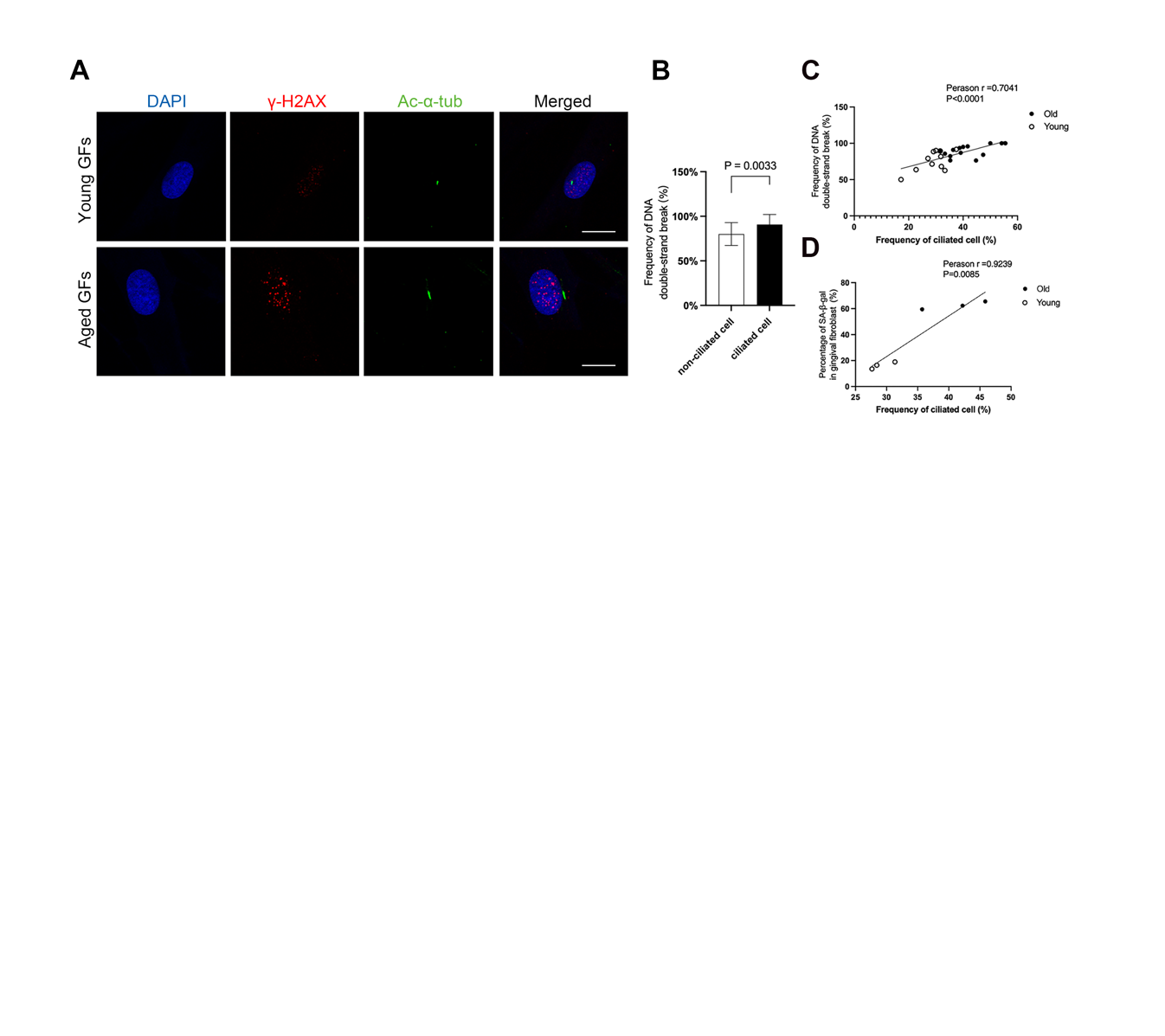
**

**Figure S4.** The linear correlation between cilia and DNA double-strand breaks.

(A) Immunofluorescence images of primary cilia and DNA damage in young and aged GFs. Green: Ac-α-tubulin; red: γ-H2AX; blue: DAPI. Scale bars: 25 μm.

(B) Quantification of DNA double-strand breaks in ciliated and non-ciliated aged GFs (n > 100 cells). Ciliated GFs exhibited a higher frequency of DNA damage.

(C) Correlation between ciliation frequency and DNA double-strand breaks (Pearson r > 0.5, n = 25).

(D) Correlation between ciliation frequency and SA-β-gal-positive cells (Pearson r > 0.8, n = 3).


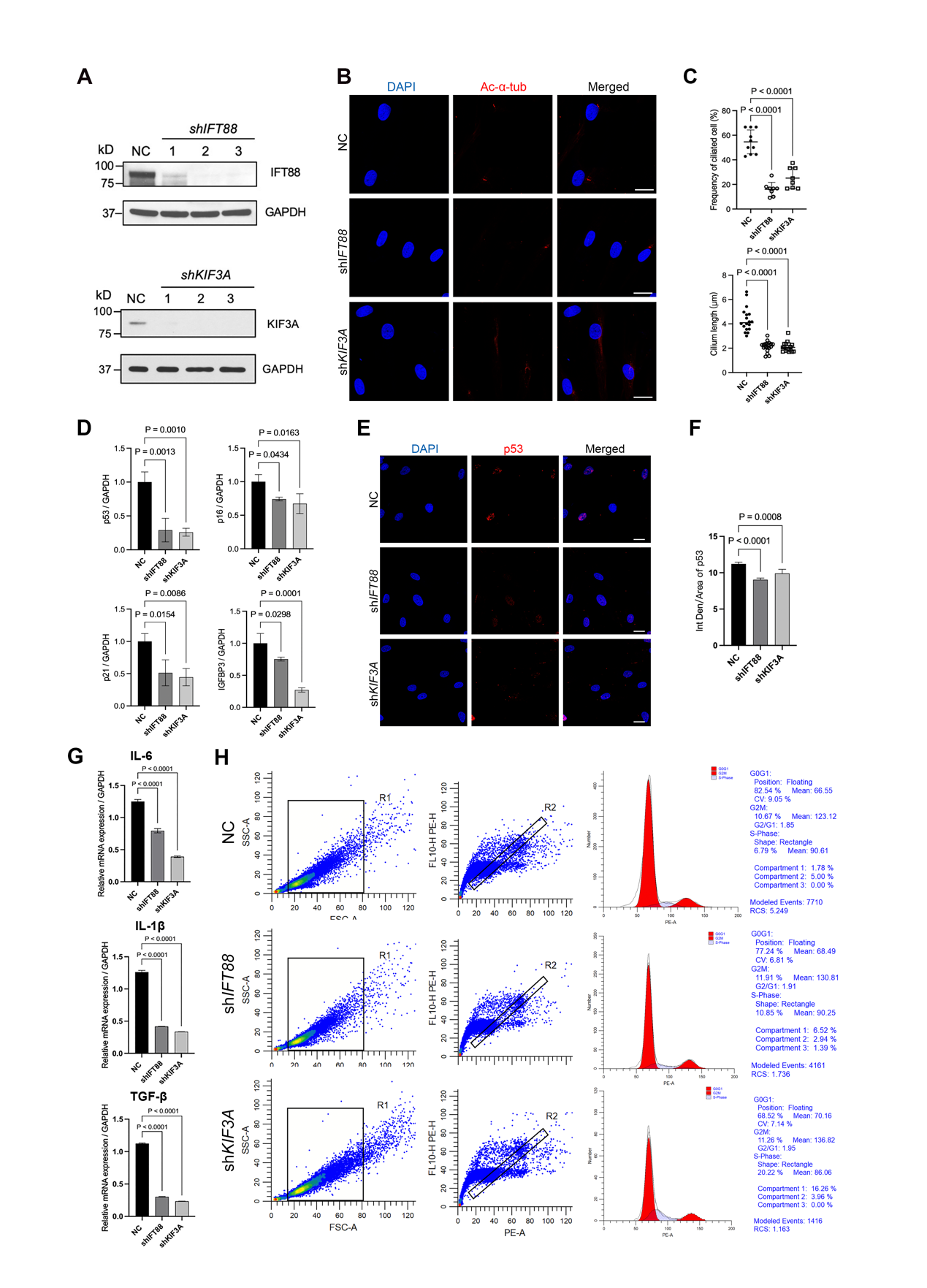


**Figure S5.** Suppression of ciliogenesis improved senescence phentotpyes in aged GFs

(A) Western blot showing IFT88 and KIF3A expression in shRNA-treated aged human GFs.

(B) Immunofluorescence of primary cilia in NC, shIFT88-, and shKIF3A-treated aged GFs. Red: Ac-α-tubulin; blue: DAPI. Scale bars: 25 μm.

(C) Quantification of ciliated cell frequency and ciliary length (n > 100 cells). Both decreased after cilia inhibition.

(D) Quantification of protein expression from Figure 4G.

(E) Immunofluorescence of p53 in NC, shIFT88-, and shKIF3A-treated aged GFs. Red: p53; blue: DAPI. Scale bars: 25 μm.

(F) Quantification of p53 fluorescence intensity (n = 3). p53 levels decreased after cilia inhibition.

(G) QRT-PCR analysis of IL-6, IL-1β, and TGF-β in NC, shIFT88-, and shKIF3A-treated GFs (n = 3). SASP gene expression declined after cilia inhibition.

(H) Flow cytometry of cell cycle phases in NC, shIFT88-, and shKIF3A-treated GFs (n = 3).

Statistical differences were analyzed using t-tests or one-way ANOVA. Data are mean ± SD (n = 3).


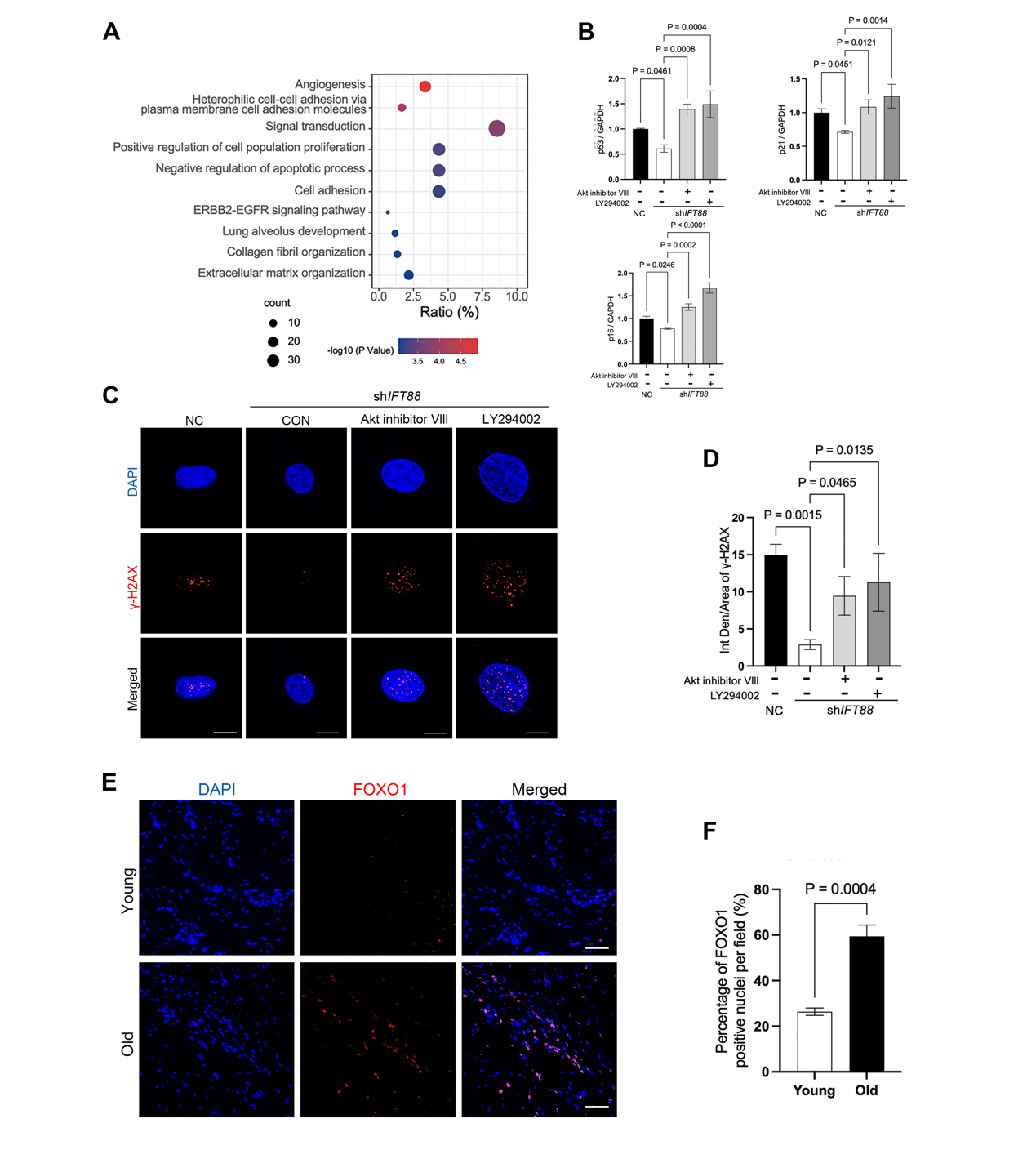


**Figure S6.** Suppression of ciliogenesis through shIFT88 and shKIF3A improved senescence phenotypes through the AKT signaling

(A) Dot plots showing representative GO pathways enriched in upregulated DEGs from shIFT88-treated aged GFs.

(B) Quantification of protein expression (n = 3) from Fig. 4E.

(C) IF of γ-H2AX in NC and shIFT88-treated GFs, with or without AKT inhibitors. Red: γ-H2AX; blue: DAPI. Scale bars: 10 μm.

(D) Quantification of γ-H2AX intensity (n = 3). DNA damage increased after AKT inhibition.

Statistical differences were analyzed using one-way ANOVA. Data are mean ± SD (n = 3).

(E-F) Immunofluorescence images of FOXO1 in young and aged human gingival tissues, with quantification of FOXO1-positive nuclei. Red: FOXO1; blue: DAPI. Scale bars: 50 μm (n = 3).


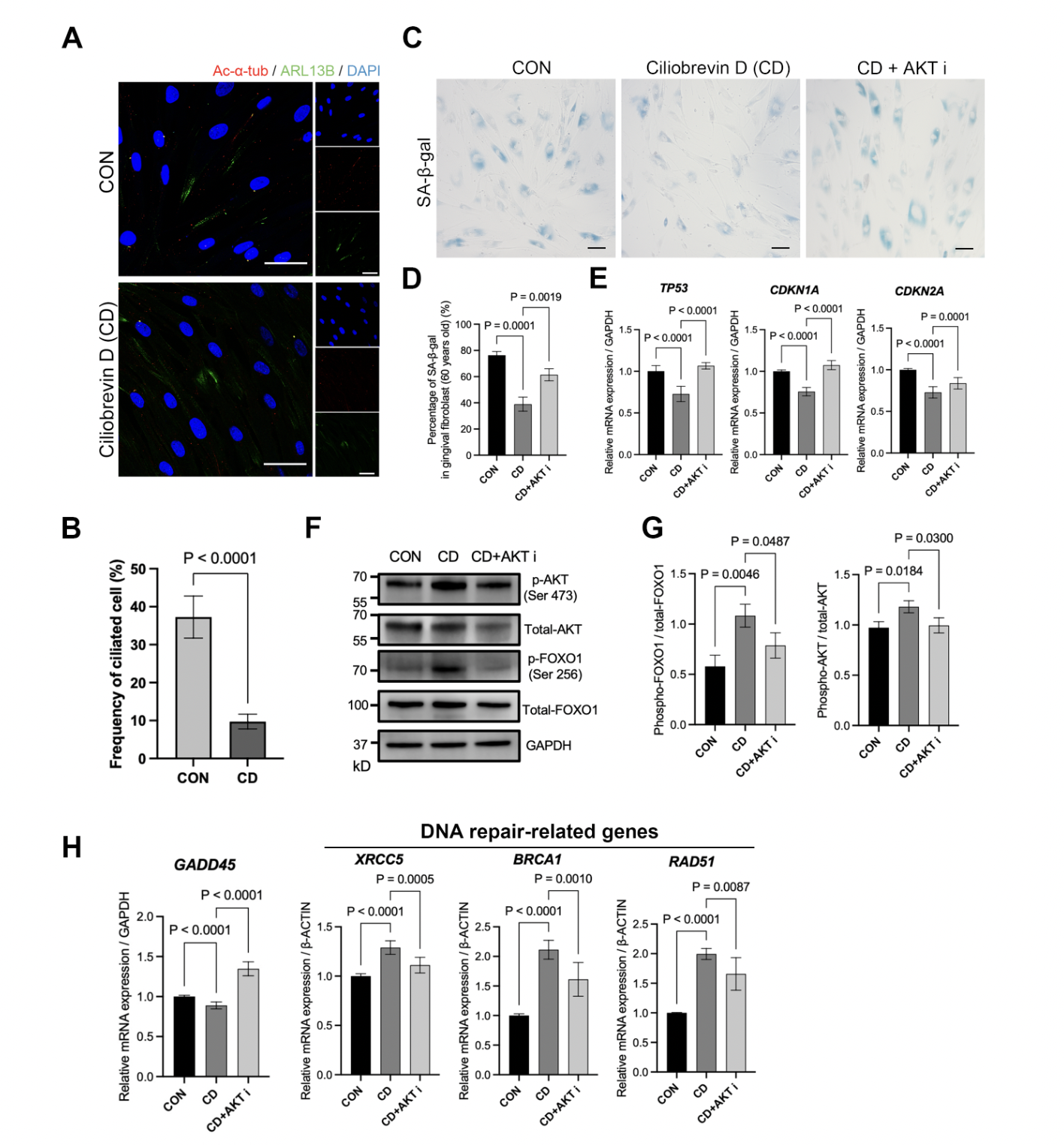


**Figure S7.** Suppressing ciliogenesis through Ciliobrevin D attenuates senescence phenotypes through the AKT signaling

(A) Immunofluorescence of primary cilia in control (CON) and Ciliobrevin D (CD)-treated aged GFs. Red: Ac-α-tubulin; Green: ARL13B; blue: DAPI. Scale bars: 25 μm.

(B) Quantification of ciliated cell frequency (n > 100) which decreased after cilia inhibition.

(C) SA-β-gal staining of control (CON) and Ciliobrevin D (CD)-treated aged GFs, with or without AKT inhibitors (AKT i). Scale bars: 100 μm.

(D) Quantification of SA-β-gal-positive cells (n = 3). Senescent cells decreased after cilia inhibition and increased following AKT inhibition.

(E) QRT-PCR analysis of TP53, CDKN1A, and CDKN2A in control and CD-treated GFs, with or without AKT inhibitors (n = 3).

(F-G) Western blot analysis of AKT phosphorylation (p-AKT) and FOXO1 phosphorylation (p-FOXO1) in control, CD-treated GFs, with or without AKT inhibitors.

(H) QRT-PCR analysis of GADD45, XRCC5, BRCA1 and RAD51 in control, CD-treated GFs, with or without AKT inhibitors (n = 3).


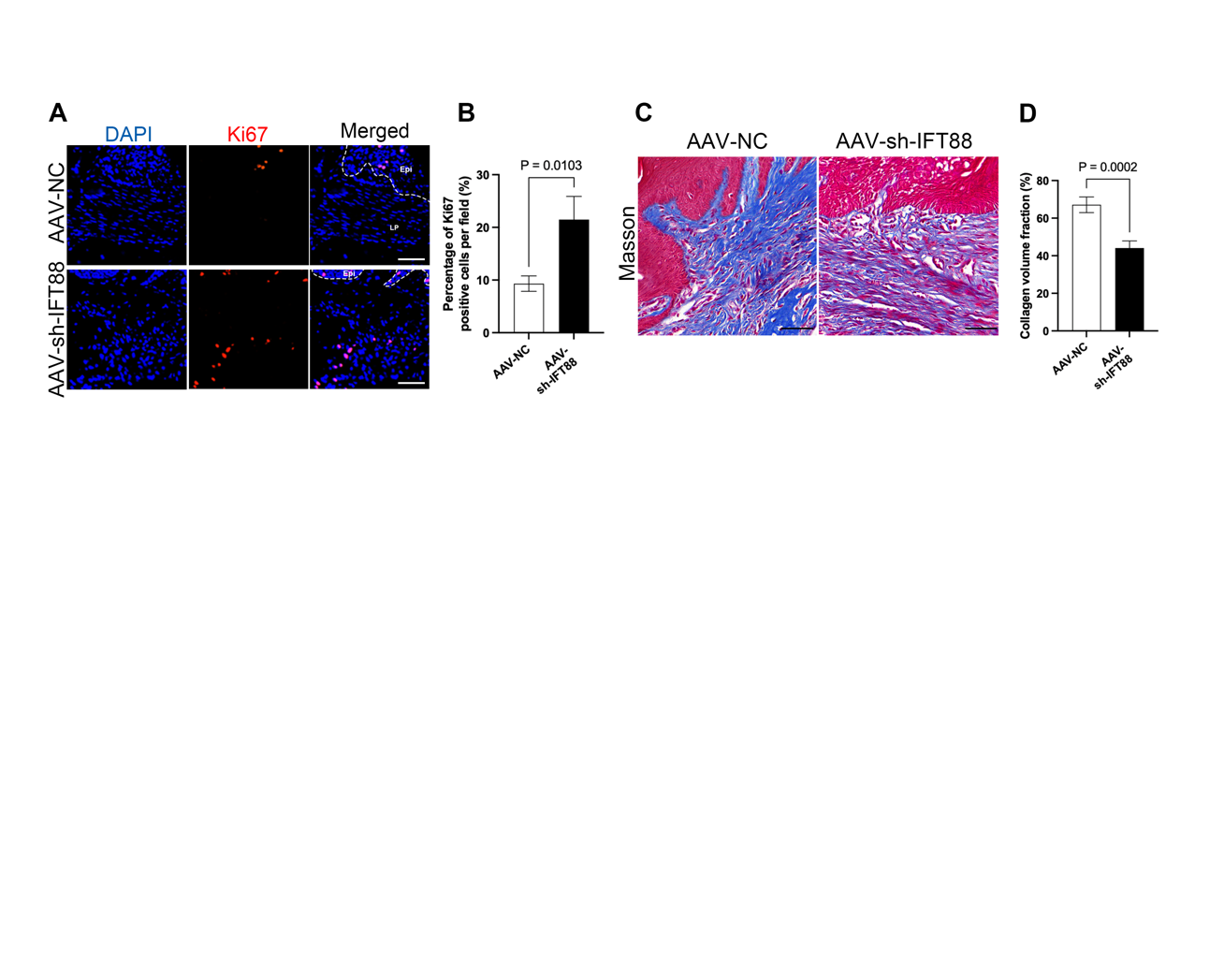


**Figure S8.** Suppressing ciliogenesis through AAV-sh-IFT88 attenuates gingival aging in mice

(A) IF images of Ki67 in gingival tissues. Red: Ki67; blue: DAPI. Scale bars: 50 μm.

(B) Quantification of Ki67-positive cells (n = 3). Proliferative cells increased after AAV-shIFT88 injection.

(C) Masson’s trichrome staining of gingival tissues. Scale bars: 25 μm.

(D) Quantification of collagen volume (n = 3). Fibrosis decreased after AAV-shIFT88 injection.

# Western Blot Images

Figure 3C

Gel percentage: 10%


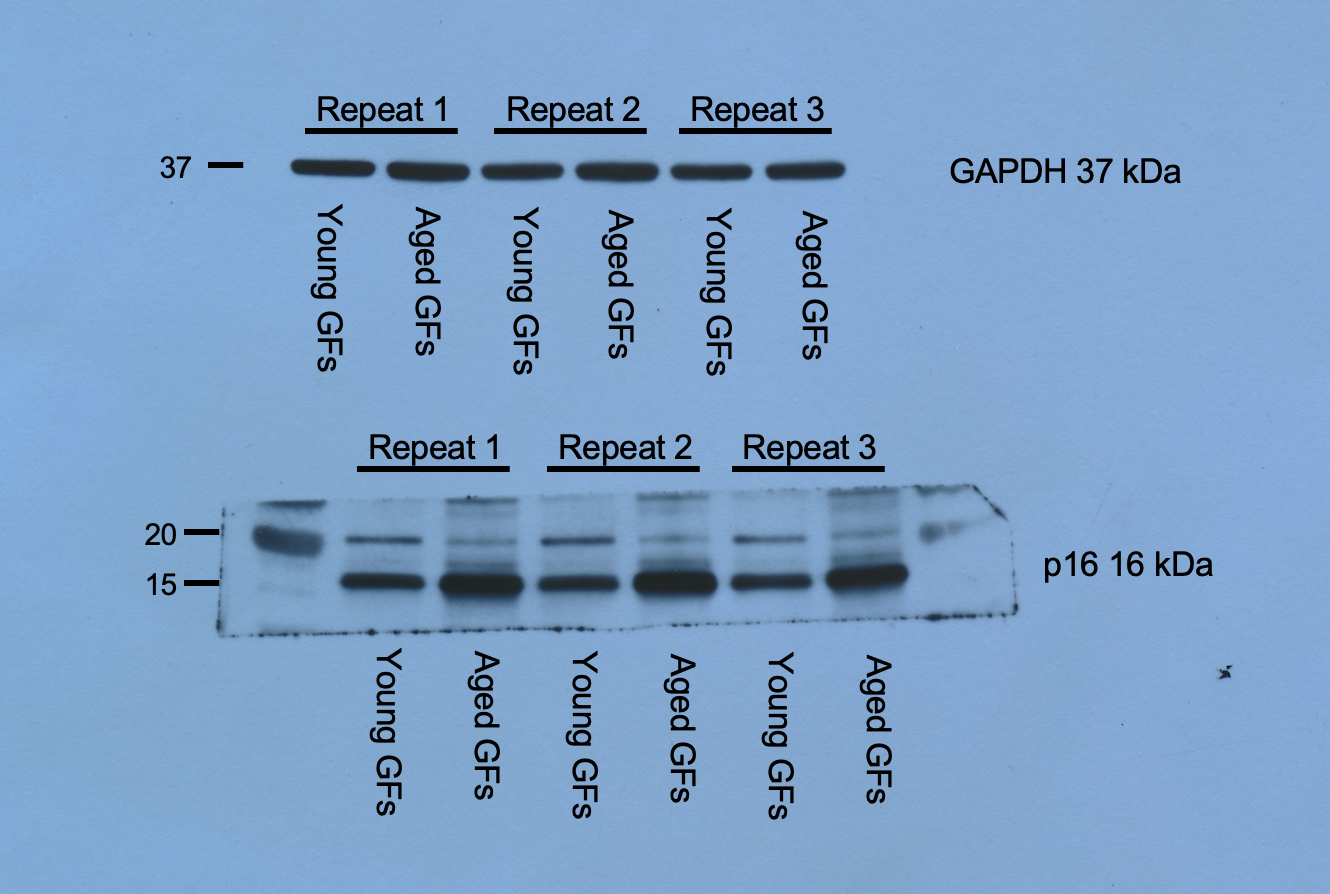


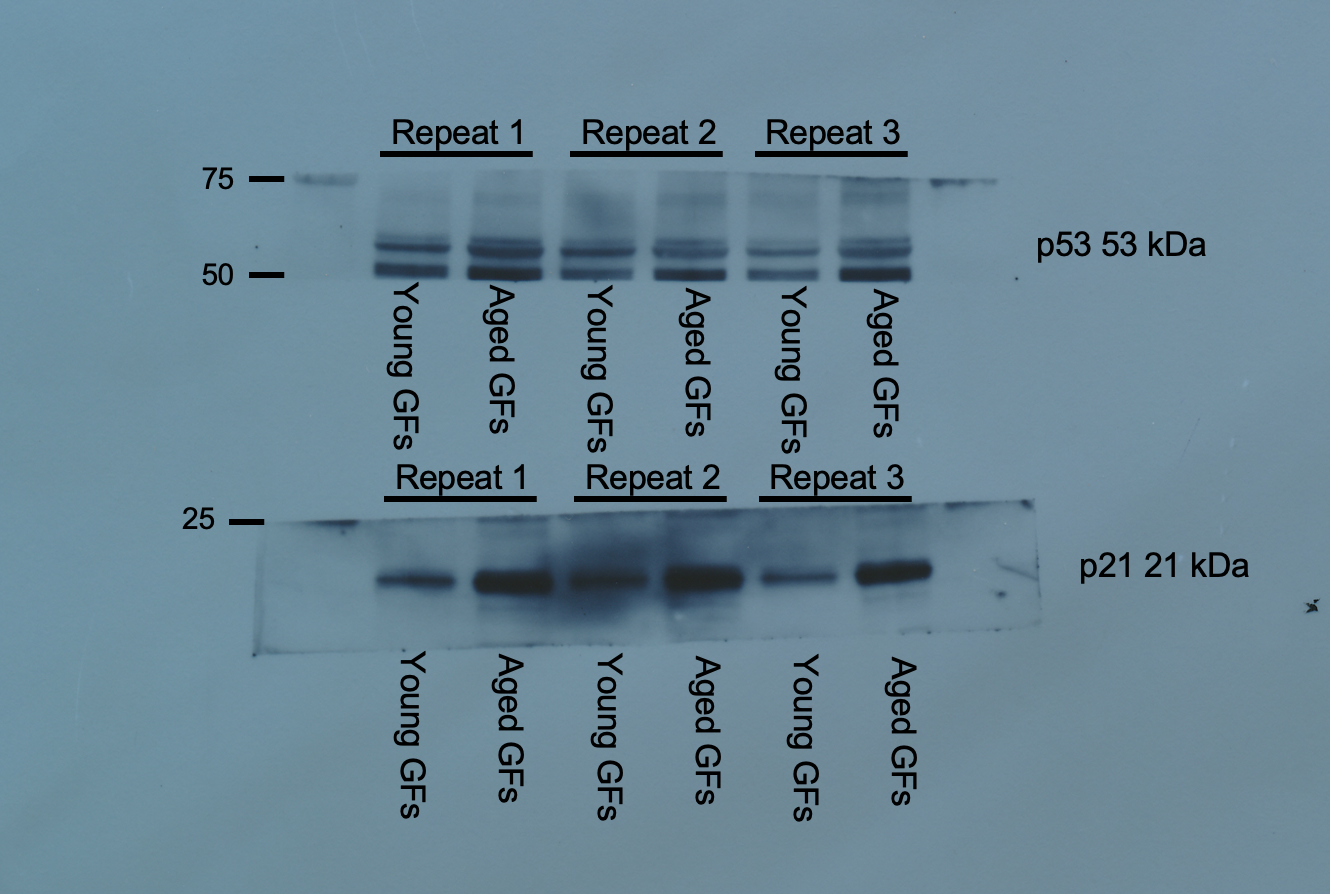

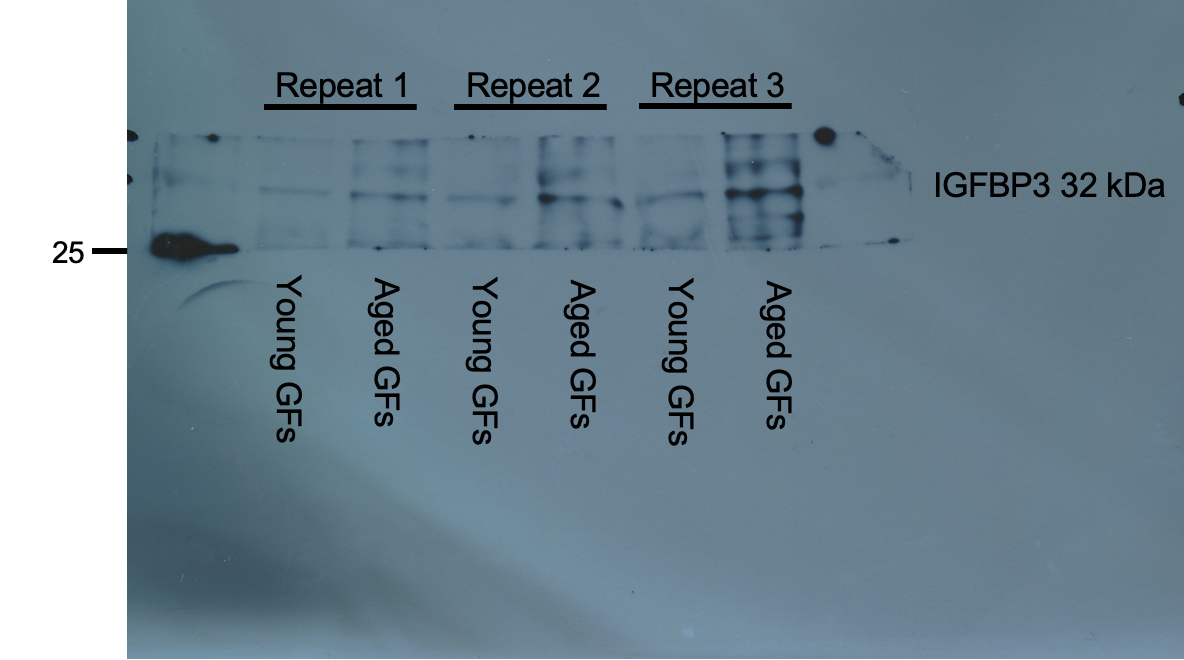


Figure 4G

Gel percentage: 10%


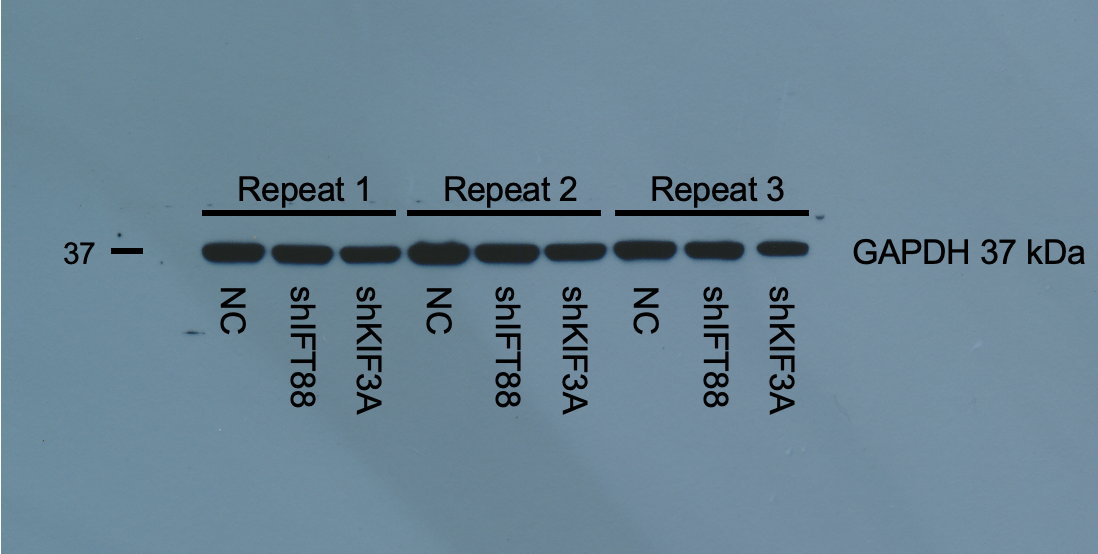


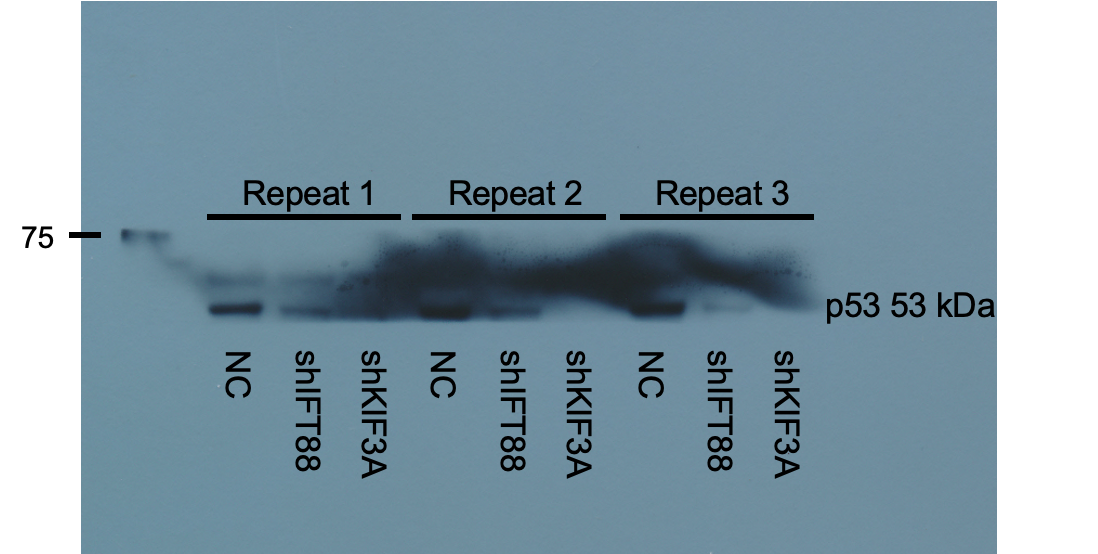


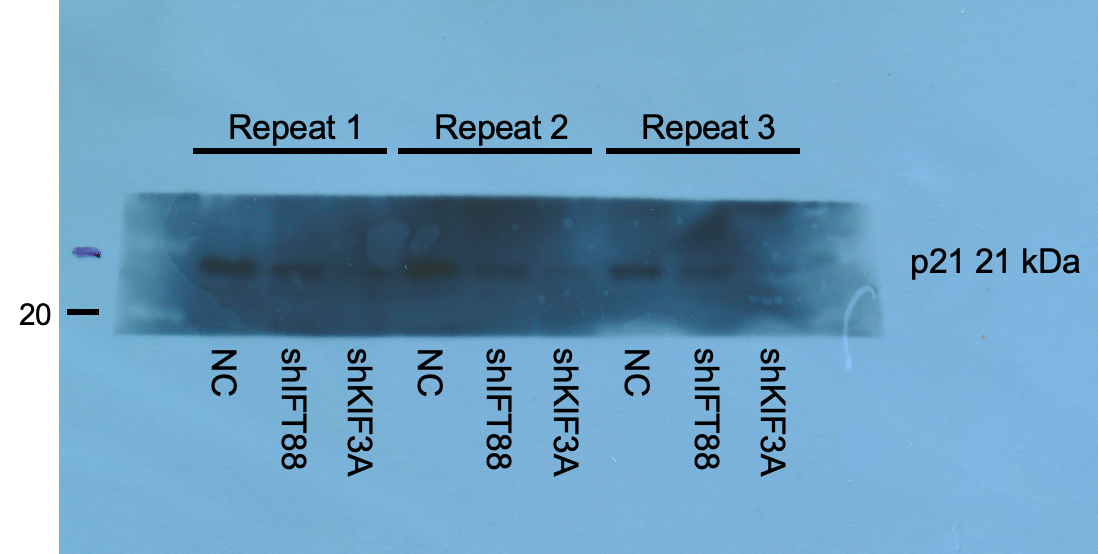


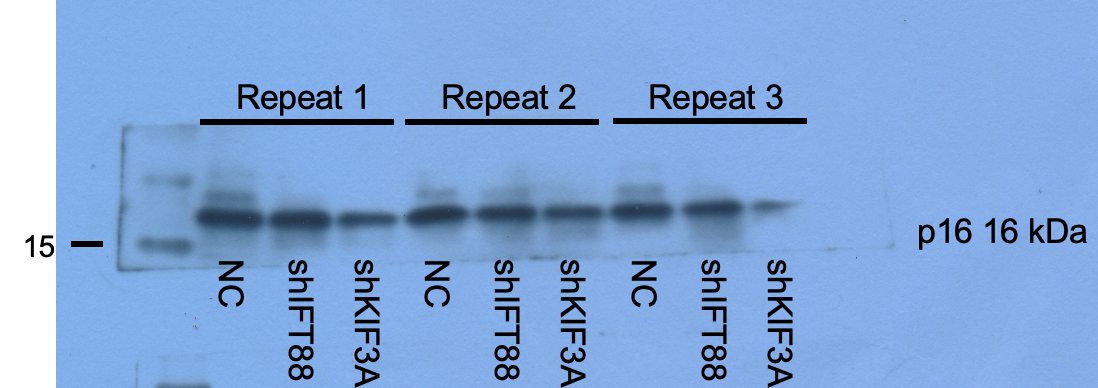


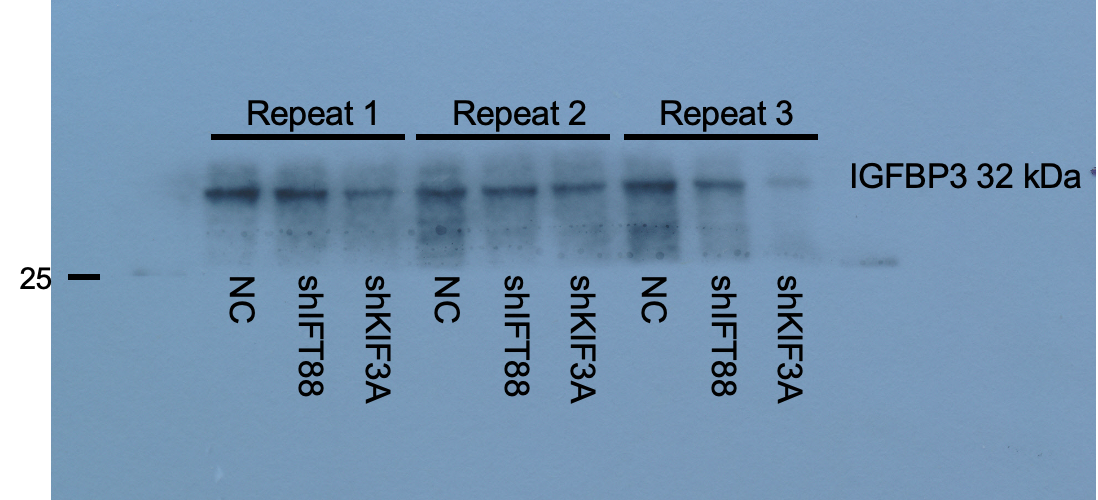


Figure 5C

Gel percentage: 10%

**
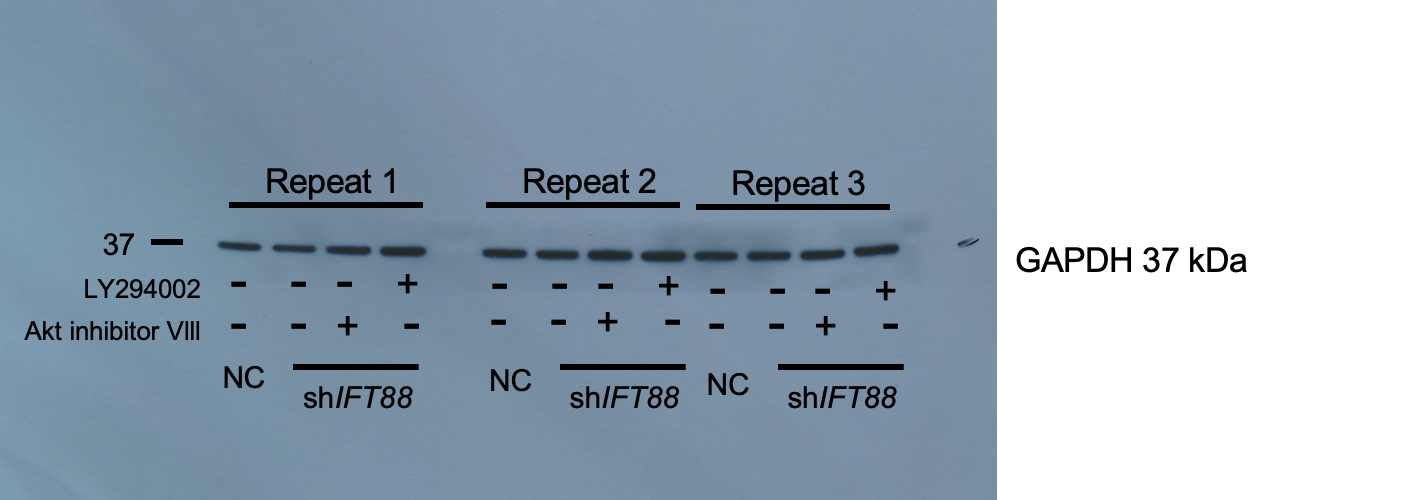
**

**
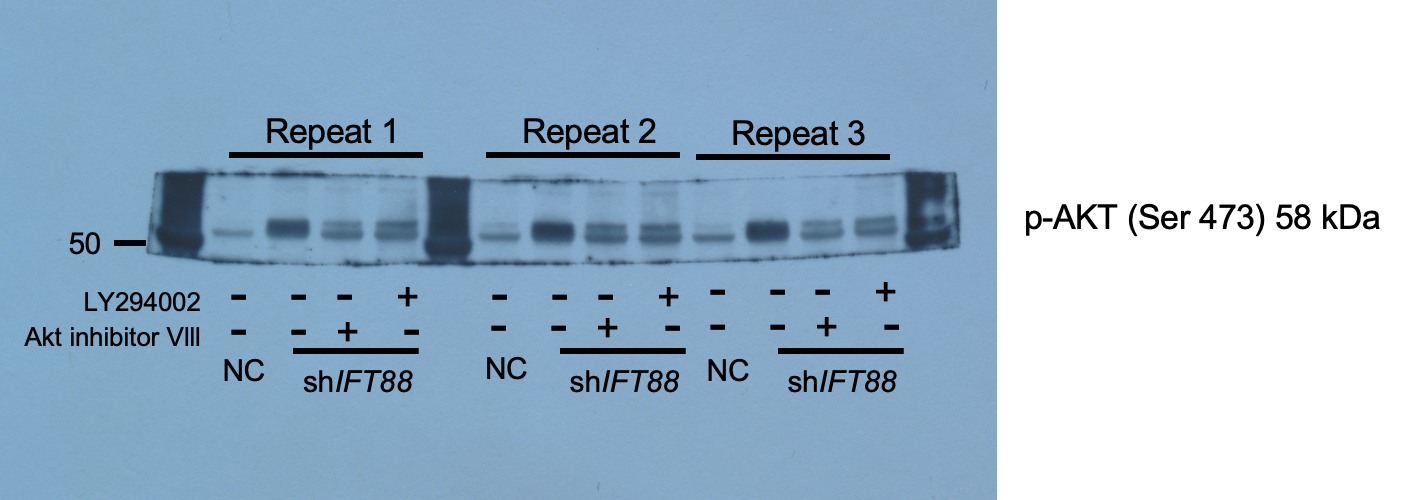
**

**
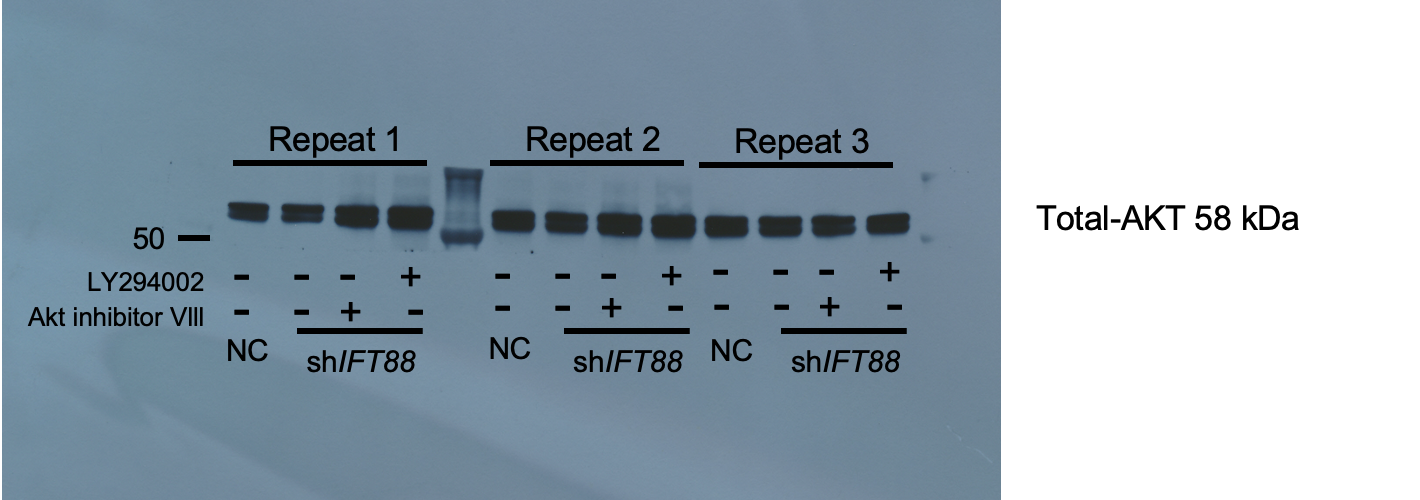
**

Figure 5E

Gel percentage: 10%


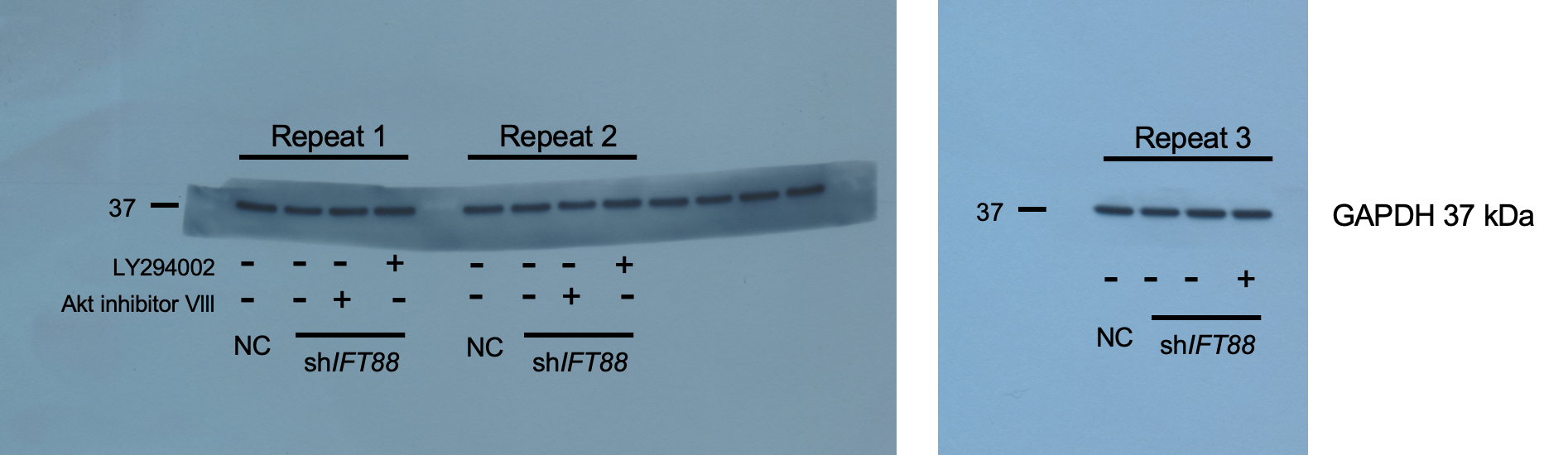


**
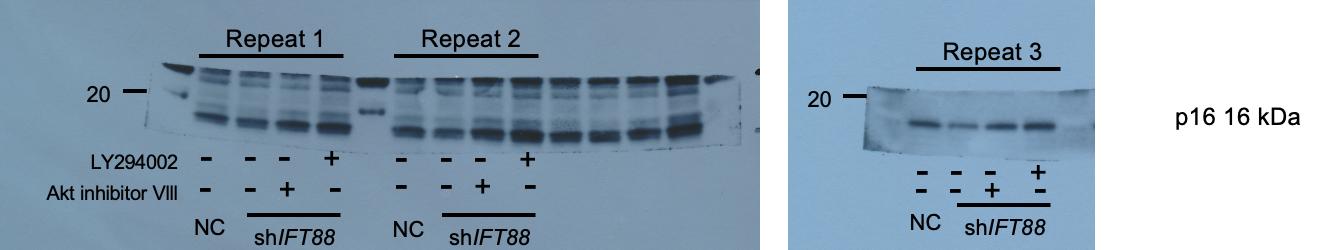
**

**
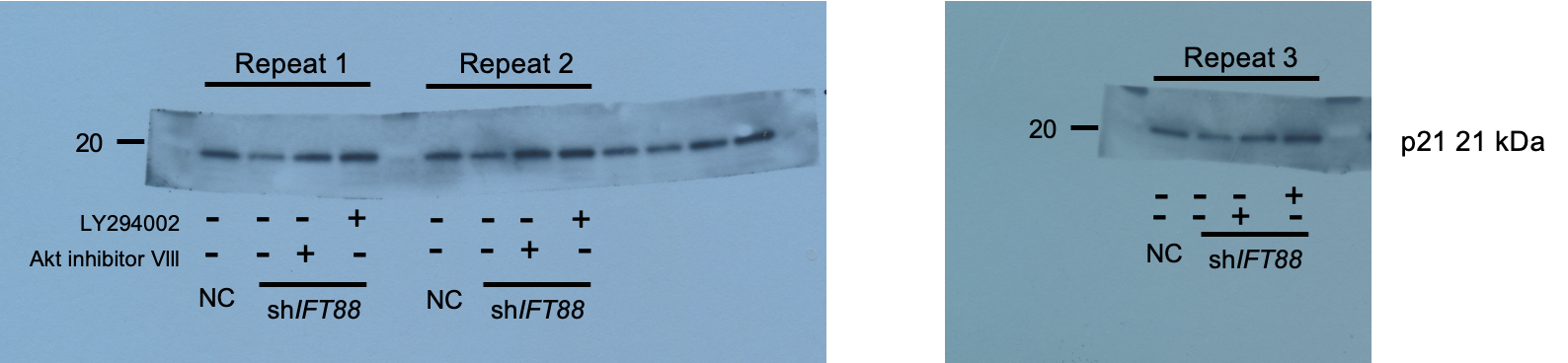
**

**
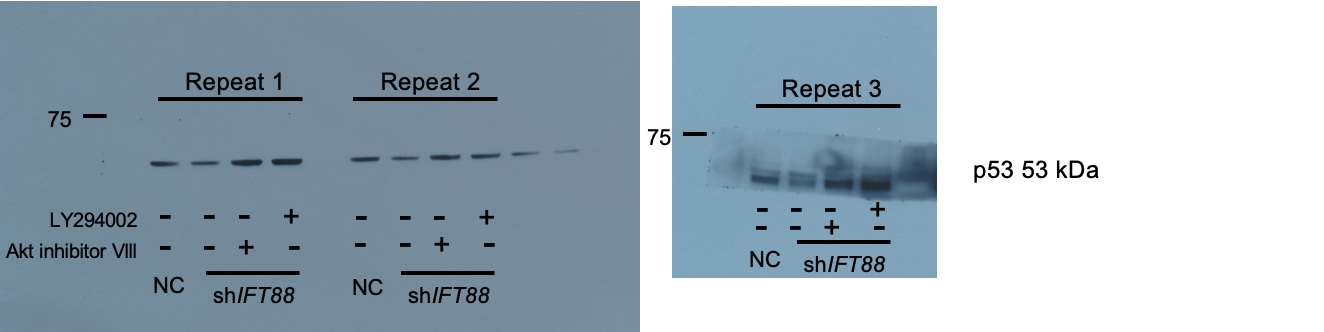
**

Figure S7F

Gel percentage: 10%


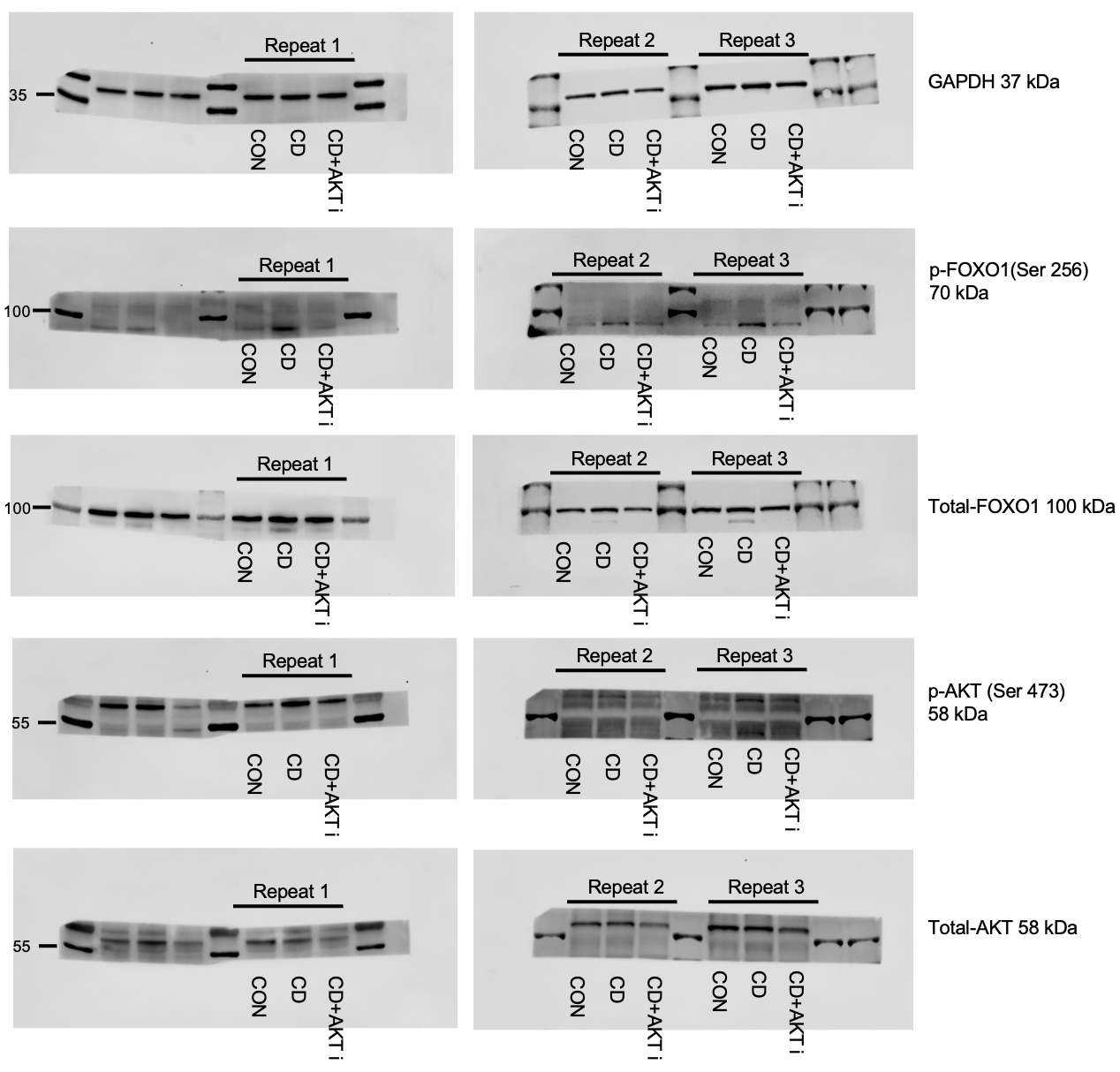

Supplement: Supplementary file 1 — Appendix S1: acel70627‐sup‐0001‐AppendixS1.zip. [file ACEL-25-e70627-s001.zip › Revised acel70627-sup-0001-AppendixS1/Supplementary File.docx]
